# Prevention of neointimal hyperplasia after coronary artery bypass graft via local delivery of sirolimus and rosuvastatin: network pharmacology and in vivo validation

**DOI:** 10.1186/s12967-024-04875-8

**Published:** 2024-02-16

**Authors:** Ji-yeon Ryu, Eui Hwa Jang, JiYong Lee, Jung-Hwan Kim, Young-Nam Youn

**Affiliations:** 1 Division of Cardiovascular Surgery, Department of Thoracic and Cardiovascular Surgery, Severance Cardiovascular Hospital, Yonsei University College of Medicine, Seoul, 03722 South Korea; 2 School of Mechanical Engineering, Yonsei University, Seoul, 03722 South Korea; 3 Department of Mechanical Engineering, University of Minnesota, Minneapolis, MN 55455 USA

**Keywords:** Sirolimus, Rosuvastatin, Intimal hyperplasia, Inflammation, Network pharmacology

## Abstract

**Background:**

Coronary artery bypass graft (CABG) is generally used to treat complex coronary artery disease. Treatment success is affected by neointimal hyperplasia (NIH) of graft and anastomotic sites. Although sirolimus and rosuvastatin individually inhibit NIH progression, the efficacy of combination treatment remains unknown.

**Methods:**

We identified cross-targets associated with CABG, sirolimus, and rosuvastatin by using databases including DisGeNET and GeneCards. GO and KEGG pathway enrichment analyses were conducted using R studio, and target proteins were mapped in PPI networks using Metascape and Cytoscape. For in vivo validation, we established a balloon-injured rabbit model by inducing NIH and applied a localized perivascular drug delivery device containing sirolimus and rosuvastatin. The outcomes were evaluated at 1, 2, and 4 weeks post-surgery.

**Results:**

We identified 115 shared targets between sirolimus and CABG among databases, 23 between rosuvastatin and CABG, and 96 among all three. TNF, AKT1, and MMP9 were identified as shared targets. Network pharmacology predicted the stages of NIH progression and the corresponding signaling pathways linked to sirolimus (acute stage, IL6/STAT3 signaling) and rosuvastatin (chronic stage, Akt/MMP9 signaling)*. *In vivo experiments demonstrated that the combination of sirolimus and rosuvastatin significantly suppressed NIH progression. This combination treatment also markedly decreased the expression of inflammation and Akt signaling pathway-related proteins, which was consistent with the predictions from network pharmacology analysis.

**Conclusions:**

Sirolimus and rosuvastatin inhibited pro-inflammatory cytokine production during the acute stage and regulated Akt/mTOR/NF-κB/STAT3 signaling in the chronic stage of NIH progression. These potential synergistic mechanisms may optimize treatment strategies to improve long-term patency after CABG.

**Graphical Abstract:**

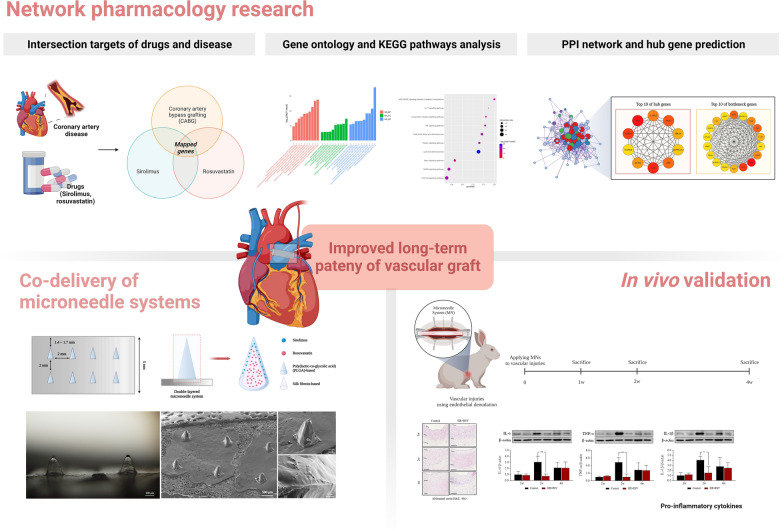

**Supplementary Information:**

The online version contains supplementary material available at 10.1186/s12967-024-04875-8.

## Background

Coronary artery disease (CAD) is a chronic and complex inflammatory disorder and it represents one of the leading causes of death worldwide [1, 2]. It is characterized by the accumulation of inflammatory factors and plaque within the coronary artery walls, which obstructs oxygen and blood flow to the heart [1, 3]. Pathogenesis of CAD entails a multifaceted interaction among genetic, lifestyle, and environmental factors, all of which exert a substantial influence on disease development and progression [4]. Therefore, a comprehensive and individualized approach is necessary to effectively address these diverse risk factors.

Coronary artery bypass graft (CABG) surgery represents the primary therapeutic option for patients with complex CAD. The aim of CABG is to improve cardiac function by redirecting blood flow around the blocked coronary arteries using autologous vessels [5]. CABG is highly effective in reducing the recurrence of cardiac events and overall mortality rates [5–7]. However, CABG treatment is often associated with challenges in maintaining long-term graft patency. Vascular trauma, when coupled with inflammation, thrombosis, or cell proliferation, can cause neointimal hyperplasia (NIH) of graft and anastomotic sites and consequent partial or total lumen obstruction. In recent studies, postoperative occlusion rates were reported to be approximately 20% for vein grafts and 5% for internal mammary artery grafts, with NIH identified as the primary common cause for both graft types [6, 8]. Consequently, these pathophysiological processes can lead to both early and late graft failure after surgery [6, 9, 10]. NIH of graft and anastomotic sites, which represents a key contributor to graft failure, is a complex process that occurs sequentially in three stages. This sequential process has significant clinical implications, particularly when it leads to vessel restenosis [6, 11]. Therefore, prevention of NIH is crucial for achieving positive long-term surgical outcomes. Although the systemic administration of drugs is commonly recommended, it provides limited local efficacy and may induce side effects. Therefore, the development of local drug delivery devices, particularly perivascular ones, has been actively studied. Application of these devices can significantly reduce systemic toxicity while maintaining a prolonged effective drug concentration at the target site [12–15].

Sirolimus, a pioneering drug in the class of mammalian target of rapamycin (mTOR) inhibitors, effectively targets the phosphoinositide 3-kinase (PI3K)/Akt/mTOR pathway. This inhibits the proliferation, migration, and transformation of vascular smooth muscle cells (VSMCs) [11, 16–18]. Owing to its effectiveness, inhibition of the mTOR pathway has become an established strategy for preventing restenosis. This has been further supported by the remarkable success of using sirolimus-eluting stents, such as Cypher® (Cordis, Bridgewater, NJ, USA), in reducing restenosis rates [17]. Furthermore, the potential clinical application of sirolimus has recently been highlighted based on the “breakthrough device designation” awarded to several sirolimus-eluting devices by the United States Food and Drug Administration [11].

Statins are β-hydroxy β-methylglutaryl-CoA reductase inhibitors, and they are considered the cornerstone of dyslipidemia treatment; their application plays a key role in the prevention and management of CAD [19, 20]. One of the fundamental advantages of statin use in preventing neointima (NI) formation involves pleiotropic effects, which are effective against disease development ranging from the hyper-acute to chronic stages. Statins have been shown to elevate nitric oxide levels during the acute stages and facilitate the degradation of the extracellular matrix (ECM) via matrix metalloproteinase (MMP) activity in the chronic stages [11, 21]. Among statins, rosuvastatin is a highly potent antihyperlipidemic drug with a broad range of pharmacodynamic activity [22]. In the context of NIH, rosuvastatin inhibits VSMC proliferation, platelet activation, and MMP-2/9 expression [23]. Although several studies have explored the local delivery effects of rosuvastatin in stents [24], research focusing on perivascular device-based delivery remains limited.

Despite the known efficacy of sirolimus and rosuvastatin in NIH prevention, evidence of their synergistic effects remains limited. Additionally, the mechanisms of their combined action when administered using a recently studied localized perivascular drug delivery device are also unclear [25]. In this context, the emerging field of network pharmacology offers valuable insights. Network pharmacology is a comprehensive approach utilized in modern pharmacological research to construct networks that elucidate drug–disease interactions, thereby facilitating the prediction of mechanisms related to drug activity [26]. The application of network pharmacology to study the combined effects of sirolimus and rosuvastatin may reveal potential synergistic mechanisms and optimize treatment strategies to improve the long-term patency after CABG.

**Fig. 1:**
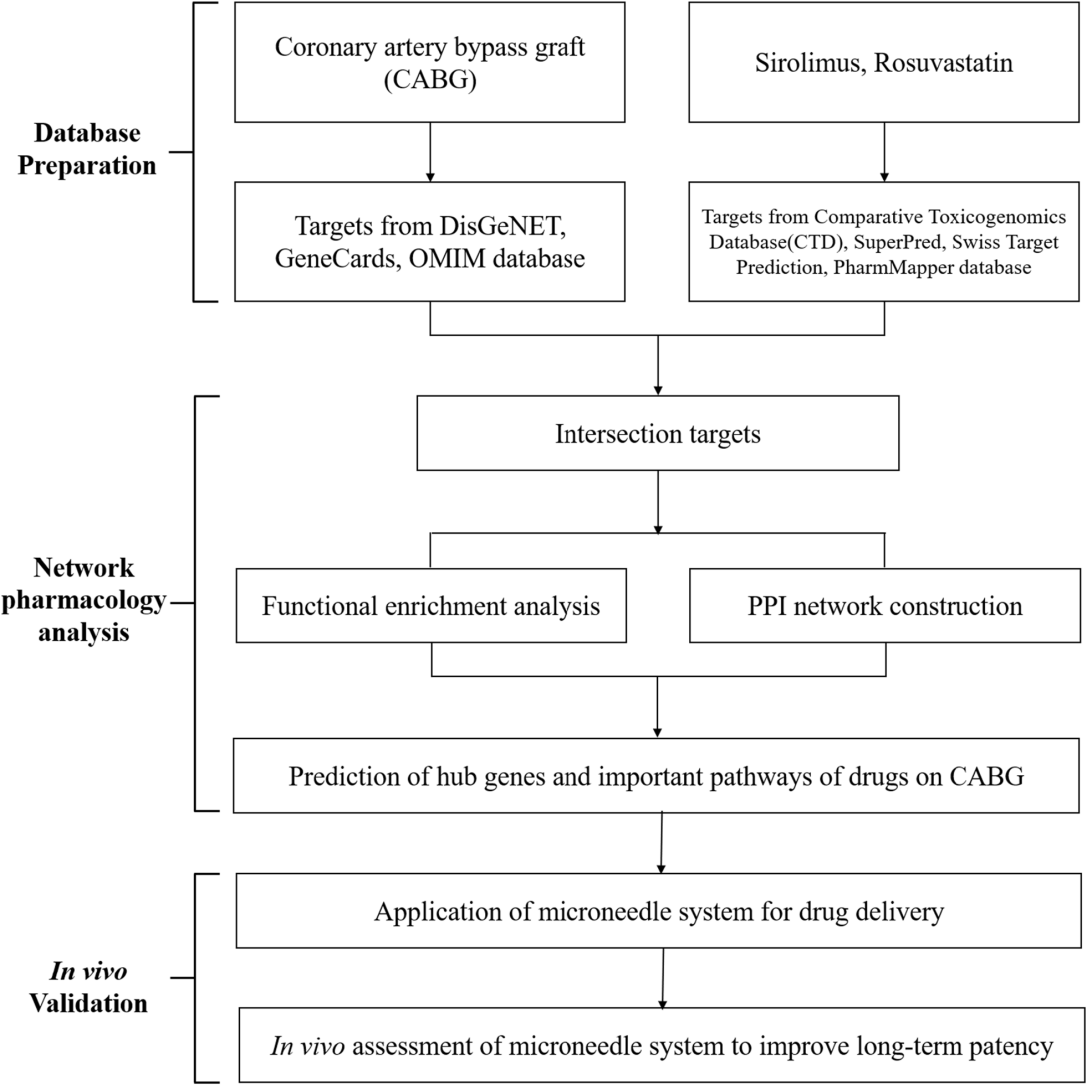
Workflow of the network pharmacology approach for identifying the mechanism underlying the effects of sirolimus and rosuvastatin in treatment coupled with CABG. *PPI* protein–protein interaction, *CABG* coronary artery bypass graft

In the present study, we used network pharmacology to explore the potential key constituents, targets, and mechanisms underlying the effects of sirolimus and rosuvastatin co-treatment coupled with CABG. We validated our findings using a balloon‐injured rabbit model and a localized perivascular drug delivery device. This research not only offers new insights that can improve long-term patency after CABG, but it also provides a scientific basis for the extensive utilization of sirolimus and rosuvastatin as potential therapeutic agents. The workflow of the study is depicted in Fig. 1.

## Materials and methods

### Network pharmacology analysis

#### Screening of CABG-related targets

CABG-related targets were predicted using DisGeNET (https://www.disgenet.org) [27], GeneCards (https://www.genecards.org) [28, 29], and OMIM (https://omics.org) database analyses [30]. The search keywords included “coronary artery bypass graft,” “intimal hyperplasia,” and “neointimal hyperplasia.” After collecting all related targets, the online tool STRING (https://string-db.org) was used to convert all targets into *Homo sapiens*-specific targets [31].

#### Screening potential therapeutic targets of sirolimus and rosuvastatin for CABG

Targets related to sirolimus and rosuvastatin were obtained from the Comparative Toxicogenomics Database (CTD, http://ctdbase.org) [32], SuperPred (https://prediction.charite.de) [33], Swiss Target Prediction (http://www.swisstargetprediction.ch) [34], and PharmMapper (http://www.lilab-ecust.cn/pharmmapper) [35]. All targets were translated into UniProt IDs using UniProtKB (https://www.uniprot.org) [36]. A Venn diagram analysis tool was subsequently used to evaluate the related targets of CABG, sirolimus, and rosuvastatin to determine their common targets for CABG treatment in conjunction with intimal hyperplasia.

#### Gene ontology (GO) and Kyoto encyclopedia of genes and genomes (KEGG) enrichment analyses

The DAVID database (https://david.ncifcrf.gov/) was utilized for conducting GO and KEGG pathway enrichment analyses [37, 38]. Results with a relatively lower -log10 value (adjusted p-value) were chosen for further analysis. The findings were visualized using R software (version 4.3.1). Bar graphs present the top 10 GO enrichment terms for biological processes (BPs), cellular components (CCs), and molecular functions (MFs), each with an adjusted p < 0.05. Bubble plots present the top 10 BPs and relevant KEGG pathways, each with an adjusted p < 0.05.

#### Construction of a protein–protein interaction (PPI) network

The intersecting targets were identified as potential therapeutic targets of sirolimus and rosuvastatin to improve the long-term patency after CABG. A PPI network was constructed, and hub genes were identified using the STRING database. Subsequently, the PPI network was visualized using Metascape (http://metascape.org/) and Cytoscape 3.9.1 (www.cytoscape.org/), which are open-source bioinformatics tools specifically developed for the visualization of molecular interaction networks [39, 40]. The identification of the top 10 hub genes was performed using the CytoHubba plug-in of Cytoscape. This analysis was based on various topological parameters, including maximal clique centrality (MCC), degree, betweenness centrality, closeness centrality, and bottleneck scores for each node [41].

### In vivo experimental validation

#### Animals

A total of 20 male New Zealand white rabbits (DooYeol Biotech, Seoul, Korea) were included in this study. The rabbits had an average weight of 3.51 ± 0.31 kg (Additional file 1: Table S1). All rabbits were maintained in accordance with the “Guide for the Care and Use of Laboratory Animals” prescribed by the National Research Council of the United States. The animal care protocol was approved by the Institutional Animal Care and Use Committee of Yonsei University Health System (Approval No. 2022-0263).

#### Chemicals and reagents

Sirolimus (#R-5000) and rosuvastatin calcium (#RHR1928) obtained from LC Laboratories (Woburn, MA, USA) and Sigma-Aldrich (St. Louis, MO, USA), respectively, were dissolved in dimethyl sulfoxide (#D0457; Samchun Chemical, Seoul, Korea). Heparin sodium was obtained from Hanlim (Seoul, Korea). Zoletil® 50 was obtained from VIRBAC (Carros, France), and aspirin was obtained from Bayer Korea (Seoul, Korea). Rhodamine B and agarose were obtained from Sigma-Aldrich.

#### Preparation and characterization of the localized perivascular drug delivery device

The localized perivascular drug delivery device was prepared as described previously [13, 42] with few modifications. Briefly, a 6.5% (w/w) silk solution was lyophilized and crystallized to fabricate porous silk wraps with a width of 10 mm and thickness of 400 μm. Simultaneously, using a microneedle mold with a height of 640 μm and an aspect ratio of 1.6, silk fibroin microneedles embedded with 2 μg of rosuvastatin calcium were fabricated and subsequently dip-coated with 1 μg of sirolimus. The sirolimus-coated and rosuvastatin-embedded silk microneedles were then arranged on a silk wrap in a 2 × 4 format. Prior to performing the in vivo experiments, all devices underwent UV sterilization for 30 min. The insertion test of the devices was performed as described previously [40]. The device and insertion site were imaged using an upright fluorescence microscope (BX40; Olympus, Tokyo, Japan), and the surface morphology and composition of the microneedles were characterized using optical microscopy and scanning electron microscopy (SEM), respectively. All illustration and characterization of the device are presented in Additional file 1: Fig. S1.

#### Rabbit abdominal aorta balloon-injured model and experimental design

All procedures, including surgery, sonography, specimen extraction, and euthanasia, were performed under general anesthesia using zoletil (10 mg/kg, intramuscularly) and isoflurane (1.5–2.0%, inhalation). We generated a balloon-injured rabbit model by inducing injury to the abdominal aorta via the femoral artery using a 2 F Fogarty® embolectomy catheter (120602F; Edwards Lifesciences, Irvine, CA, USA). Following injury, the rabbits were segregated into two groups (n = 3–5/group). One group was treated using a perivascular drug delivery device lacking any drug (control), whereas the other group was treated using a device containing sirolimus and rosuvastatin (SIR + RSV). The perivascular drug delivery device was carefully wrapped around the external surface of the injured abdominal aorta and secured using a surgical clip (LIGACLIP^®^, Titanium Medium, Mexico). The clip was adjusted to match the individual aortic diameter, which was preoperatively measured using ultrasonography. To prevent acute thrombosis, heparin sodium (100 U/kg) was administered immediately before inducing balloon injury, and aspirin (100 mg/day) was administered for 4 weeks post-surgery.

#### Histological analysis

Histopathological evaluation was conducted using hematoxylin and eosin (H&E) staining. Samples were obtained at specific time points (1, 2, and 4 weeks after surgery) for the purpose of histological analysis. The collected samples were preserved in a solution of 10% buffered formalin. Subsequently, cross-sections with a thickness of 5 µm were prepared and then embedded in paraffin. The morphological changes in different layers of blood vessels and the progression of NIH were analyzed and confirmed using H&E staining. The structural characteristics of each histological section were determined via observation under an OLYMPUS BX53 light microscope. Measurements of the lumen area, as well as the intima and media thickness, were conducted using ImageJ software (version 1.52v; National Institutes of Health, Bethesda, MD, USA). The ratio of intima-to-media was subsequently calculated.

#### Transmission electron microscopy (TEM)

Abdominal aortic tissue samples were obtained from the experimental subjects and subsequently sectioned into 1-mm square fragments. The specimens were immersed in a solution of 2% buffered glutaraldehyde and subsequently preserved in the same fixative before storage in a refrigerator at 4 °C. Following the standard fixation procedure, the tissues were rinsed and dehydrated using graded ethanol and phosphate buffer series. The tissue samples were subsequently embedded using a Poly/Bed 812 kit (Polysciences, Warrington, PA, USA). The tissue sections were sliced into 200-nm semi-thin sections using a diamond knife on an ultramicrotome. Subsequently, the sections were stained with toluidine blue to facilitate observation under an optical microscope. The samples underwent analysis and imaging using a transmission electron microscope (JEM-1011; JEOL, Tokyo, Japan) operated at an acceleration voltage of 80 kV. The microscope was equipped with a MegaView III CCD camera (Soft Imaging System, Germany).

#### Western blotting

Western blotting was conducted following the protocol outlined by Song et al*.* (2018), with minor modifications [43]. The primary antibodies used in this study were interleukin 6 (IL-6), tumor necrosis factor alpha (TNF-α), signal transducer and activator of transcription 3 (STAT3), phosphorylated STAT3 (p-STAT3), AKT serine/threonine kinase 1 (Akt1), mTOR, phosphorylated mTOR (p-mTOR), nuclear factor (NF)-κB, MMP9 (all at a dilution of 1:500; Santa Cruz Biotechnology, Santa Cruz, CA, USA), IL-1β (1:500; Cell Signaling Technology, Danvers, MA, USA), and anti-β-actin antibody (1:10,000; Cell Signaling Technology). A horseradish peroxidase-conjugated goat anti-mouse immunoglobulin G secondary antibody (1:5,000; GenDEPOT, Katy, TX, USA) was used to visualize the blots. The blots were visualized using West-Q Pico Dura and Femto ECL solution (GenDEPOT).

### Statistical analysis

The experiments were conducted in triplicate at least, and the data are presented as the mean ± standard deviation. Statistical analyses were conducted using GraphPad Prism 10.0 software (GraphPad Software, La Jolla, CA, USA) and SPSS software (version 26.0; SPSS, Chicago, IL, USA). The differences between groups were analyzed using one-way analysis of variance.

## Results

### Screening potential therapeutic targets of sirolimus and rosuvastatin for CABG treatment

**Fig. 2:**
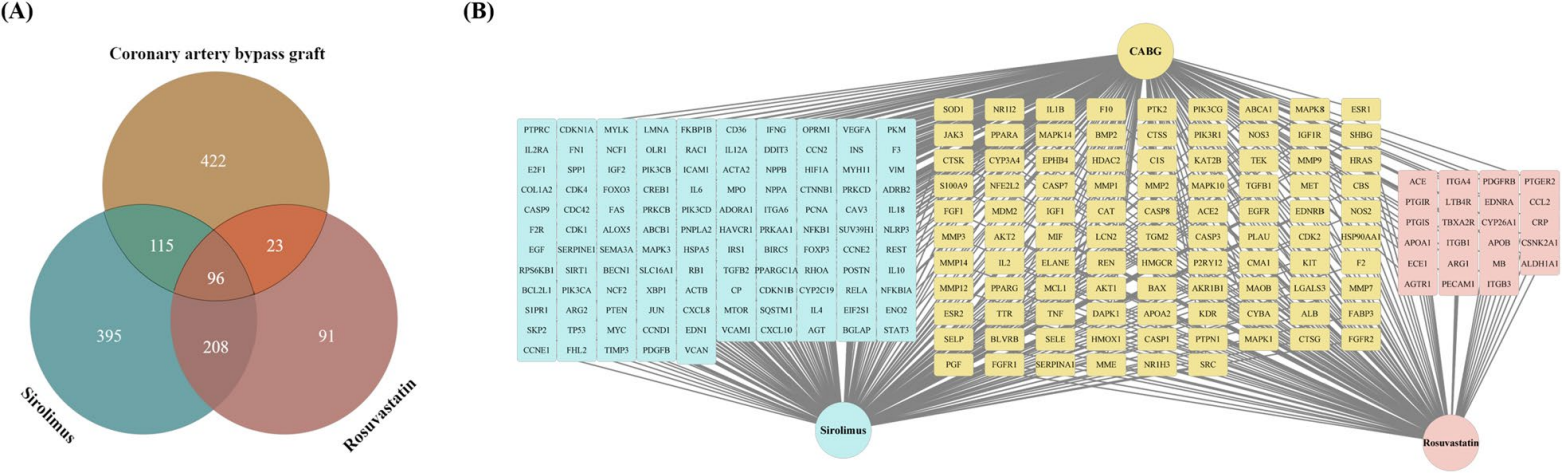
Intersection of potential targets of CABG, sirolimus, and rosuvastatin along with overlapping targets in a drug-disease network. **A** Venn diagram showing the potential targets of CABG, sirolimus, and rosuvastatin. Yellow section represents the potential targets of CABG, blue section represents the potential targets of sirolimus, and pink section represents the potential targets of rosuvastatin. **B** Overlapping targets in the drug-disease network. CABG, coronary artery bypass graft

To identify the intersecting genes and construct drug-drug and drug-disease networks, we compiled lists of genes related to CABG and the drugs sirolimus and rosuvastatin. We screened the DisGeNET, GeneCards, and OMIM databases for CABG-related disease targets and identified 656 unique targets. Redundancies were excluded using the UniProt database. Subsequently, from the CTD, SuperPred, Swiss Target Prediction, and PharmMapper databases, we assembled 815 and 417 pharmacological targets for sirolimus and rosuvastatin, respectively. Redundant genes were filtered out using the UniProt database. Venn diagram analysis showed that sirolimus and CABG had 115 shared targets, rosuvastatin and CABG had 23 shared targets, and all three had 96 shared targets (Fig. 2A).

To directly present the network topology status of sirolimus and rosuvastatin for CABG, we constructed a shared target network. In the network shown, blue nodes represent shared targets between sirolimus and CABG, pink nodes indicate shared targets between rosuvastatin and CABG, and yellow nodes represent shared targets among all three, such as TNF, AKT1, and MMPs (Fig. 2B).

### Exploring the therapeutic potential of sirolimus and rosuvastatin against NIH using GO and KEGG enrichment analysis

**Fig. 3:**
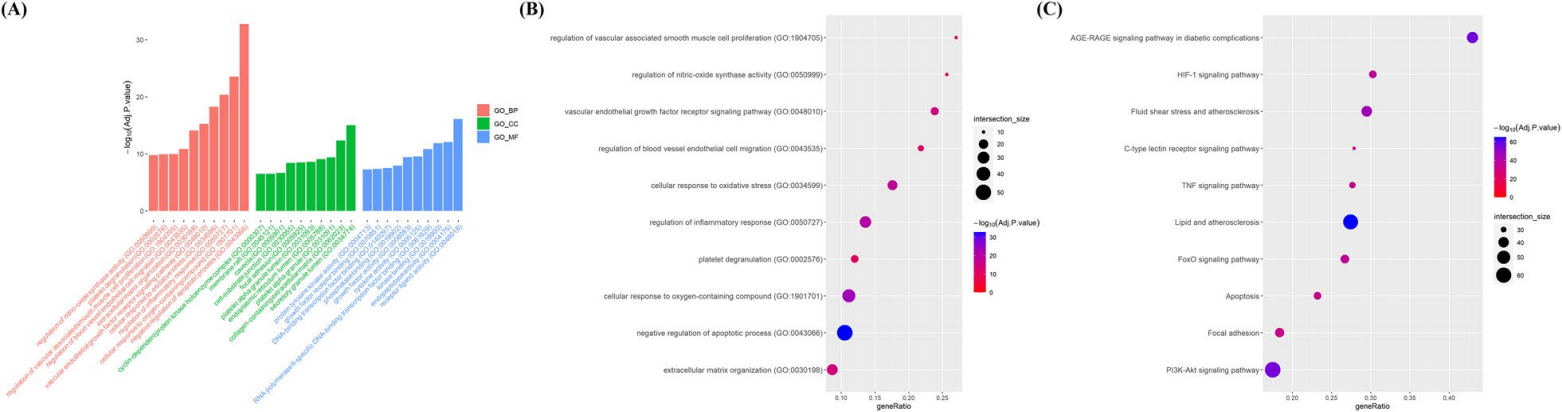
GO and KEGG pathway analysis of overlapping targets between CABG and sirolimus. **A** Bar graph presents the top 10 significant GO terms related to vascular disease among overlapping targets. X-axis presents the top 10 BPs, CCs, and MFs identified via GO enrichment analysis. Y-axis presents the − log10 (adjusted p-value) identified within each GO term. Dot plots illustrating the enrichment of **B** BP and **C** KEGG pathways. X-axis presents the enrichment gene ratio, while y-axis presents the BP and KEGG pathways. Dot size indicates the number of genes, and color indicates the enriched − log10 (adjusted p-value). *GO* gene ontology, *KEGG* Kyoto encyclopedia of genes and genomes, *CABG* coronary artery bypass graft, *BP* biological process, *CC* cellular component, *MF* molecular function

**Fig. 4:**
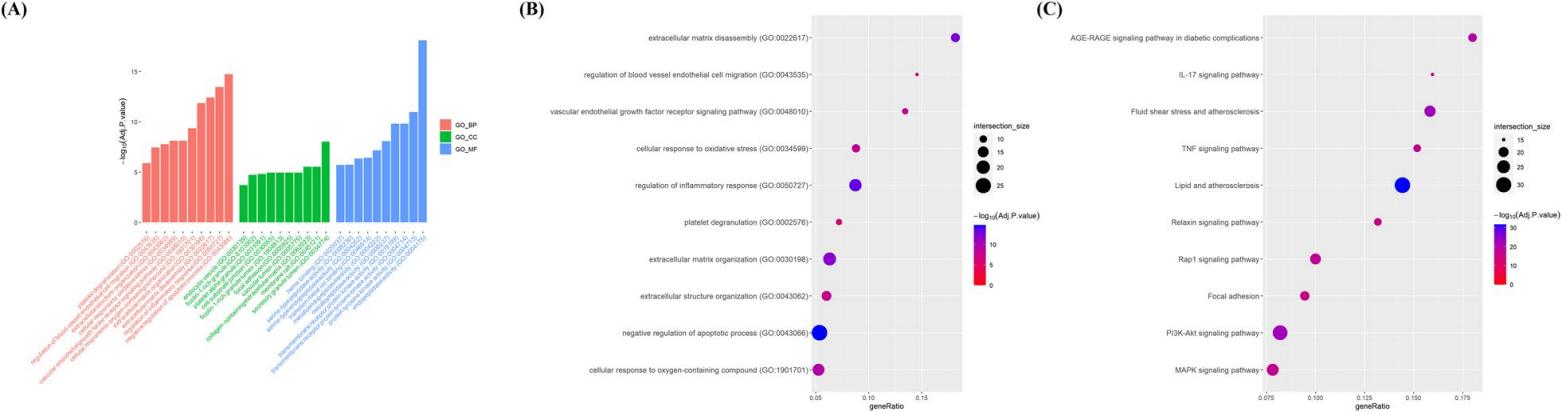
GO and KEGG pathway analysis of overlapping targets between CABG and rosuvastatin. **A** Bar graph presents the top 10 significant GO terms related to vascular disease among overlapping targets. X-axis presents the top 10 BPs, CCs, and MFs identified via GO enrichment analysis. Y-axis presents the − log10 (adjusted p-value) identified within each GO term. Dot plots illustrating the enrichment of **B** BP and **C** KEGG pathways. X-axis presents the enrichment gene ratio, while y-axis presents BP and KEGG pathway. Dot size indicates the number of genes, and color indicates the enriched − log10 (adjusted p-value). *GO* gene ontology, *KEGG* Kyoto encyclopedia of genes and genomes, *CABG* coronary artery bypass graft, *BP* biological process, *CC* cellular component, *MF* molecular function

**Fig. 5:**
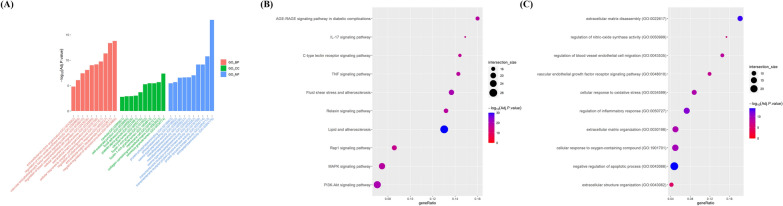
GO and KEGG pathway analysis of overlapping targets among CABG, sirolimus, and rosuvastatin. **A** Bar graph presents the top 10 significant GO terms related to vascular disease among overlapping targets. X-axis presents the top 10 BPs, CCs, and MFs identified via GO enrichment analysis. Y-axis presents the − log10 (adjusted p-value) identified within each GO term. Dot plots illustrating the enrichment of **B** BP and **C** KEGG pathways. X-axis presents the enrichment gene ratio, while y-axis presents BP and KEGG pathways. Dot size indicates the number of genes, and color represents the enriched − log10 (adjusted p-value). *GO* gene ontology, *KEGG* Kyoto encyclopedia of genes and genomes, *CABG* coronary artery bypass graft, *BP* biological process, *CC* cellular component, *MF* molecular function

To further evaluate the intersecting targets associated with NIH, we performed GO and KEGG enrichment analyses. We conducted analyses for each group of shared targets: sirolimus and CABG, rosuvastatin and CABG, and the combination of sirolimus, rosuvastatin, and CABG. We subsequently selected and visualized the top 10 categories from each of the three domains—BP, CC, and MF—as well as the KEGG pathways (Fig. 3, 4, 5).

#### GO and KEGG enrichment analysis for sirolimus and CABG

The shared targets of sirolimus and CABG were found to be significantly enriched in BPs and pathways related to the regulation of apoptotic process, regulation of inflammatory response, endothelial cell migration, and VSMC proliferation (Fig. 3A and B, Additional file 1: Tables S2–S4). KEGG enrichment analysis showed that these shared targets were strongly linked to the PI3K-Akt signaling pathway, fluid shear stress/lipid and atherosclerosis, and the TNF signaling pathway (Fig. 3C and Additional file 1: Table S5).

#### GO and KEGG enrichment analysis for rosuvastatin and CABG

GO enrichment analysis of shared targets between rosuvastatin and CABG showed significant enrichment in negative regulation of apoptotic process, regulation of inflammatory response, and ECM disassembly and organization (Fig. 4A and B, Additional file 1: Tables S6–S8). KEGG enrichment analysis demonstrated that the potential targets were primarily associated with pathways, such as PI3K-Akt, mitogen-activated protein kinase (MAPK), TNF, and IL-17 (Fig. 4C and Additional file 1: Table S9).

#### GO and KEGG enrichment analysis for combined sirolimus, rosuvastatin, and CABG

We found that sirolimus, rosuvastatin, and CABG have 96 shared targets that are significantly linked to various biological activities, including the negative regulation of apoptotic process, regulation of inflammatory response, ECM disassembly and organization, cellular oxidative stress, and pathways, such as PI3K-Akt, MAPK, TNF, IL-17, fluid shear stress, lipid and atherosclerosis (Fig. 5 and Additional file 1: Tables S10–S13). These results help explain the changes in biological function and related pathways when sirolimus and rosuvastatin are used either alone or in combination for NIH treatment after CABG.

### *Predicting CABG-related hub genes for sirolimus and rosuvastatin *via* PPI network analysis*

**Fig. 6:**
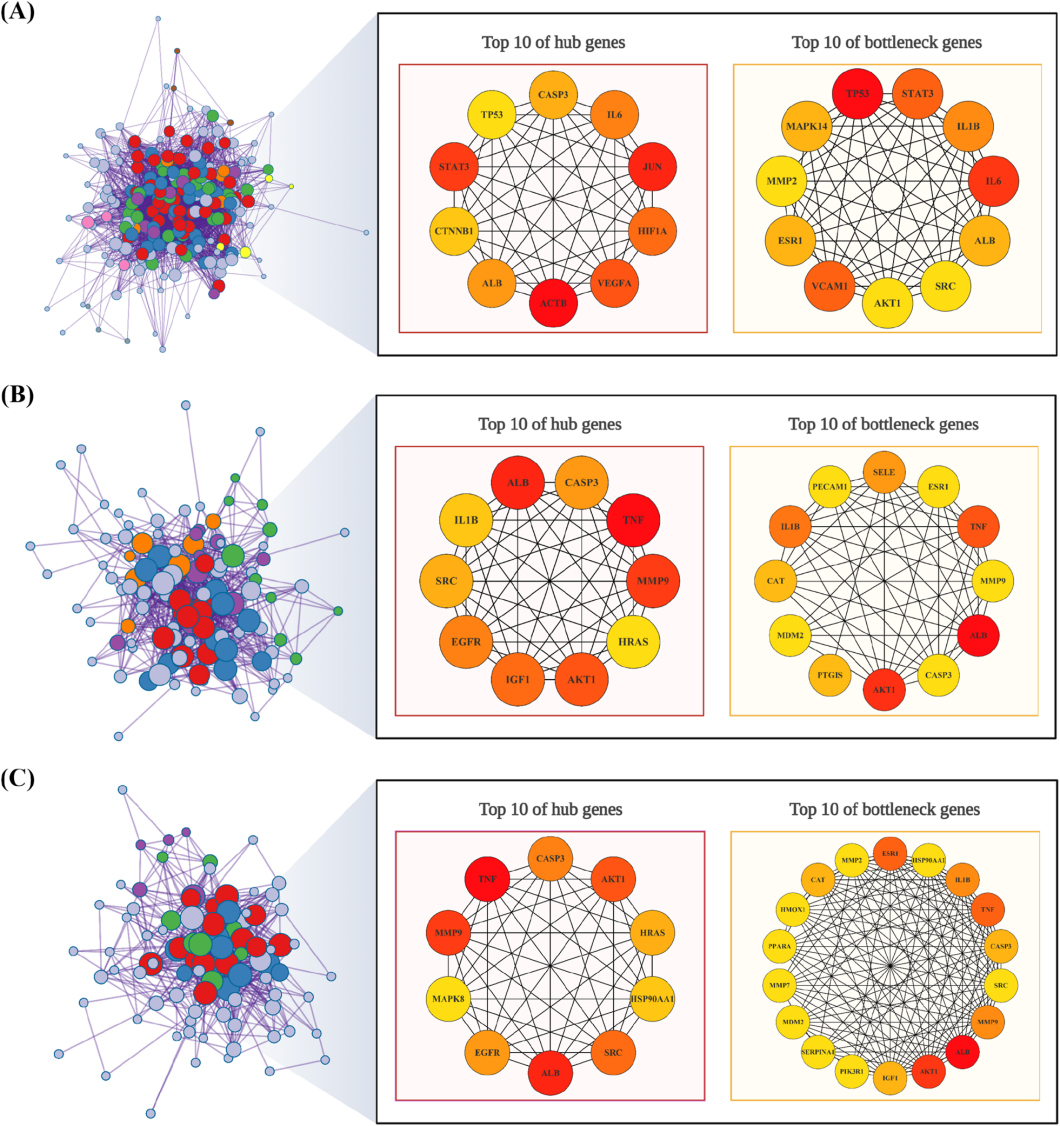
Prediction of the drug combination of sirolimus and rosuvastatin for CABG via PPI network analysis. **A**–**C** The interactive PPI network of **A** CABG and sirolimus, **B** CABG and rosuvastatin, and **C** CABG and both drugs. The top 10 hub and bottleneck genes identified using CytoHubba are shown. Dark red color indicates a relatively higher maximal clique centrality or bottleneck score. The figures were created using BioRender. *CABG* coronary artery bypass graft, *PPI* protein–protein interaction

We examined the hub genes and potential mechanisms related to the use of sirolimus and rosuvastatin in the treatment of NIH of graft and anastomotic sites after CABG. The STRING database was used to construct PPI networks for shared targets of sirolimus, rosuvastatin, and CABG. We used the Metascape and Cytoscape software to visualize the network. Additionally, topological parameters were calculated. We used the CytoHubba plug-in of Cytoscape to identify hub and bottleneck genes in the PPI network, using MCC and bottleneck scores (Fig. 6; Table 1 and Additional file 1: Table S14).

**Table 1:** Top 10 significant core targets ranked using CytoHubba

No	Name	Description	MCC Score	BottleNeck Score	DC	BC	CC
Sirolimus							
1	TP53^*^	Tumor protein p53	9.22E + 13	50.00	146.00	972.93	178.00
2	IL6^*^	Interleukin 6	9.22E + 13	19.00	163.00	1,400.41	186.50
3	STAT3^*^	Signal transducer and activator of transcription 3	9.22E + 13	15.00	134.00	541.92	172.00
3	VCAM1	Vascular cell adhesion molecule 1	9.22E + 13	15.00	87.00	194.11	148.33
5	IL1B	Interleukin 1 beta	9.22E + 13	8.00	146.00	898.27	178.00
6	MAPK14	Mitogen-activated protein kinase 14	9.22E + 13	6.00	93.00	214.59	151.50
6	ESR1	Estrogen receptor 1	9.22E + 13	6.00	113.00	489.63	161.33
6	ALB^*^	Albumin	9.22E + 13	6.00	161.00	1,908.35	185.50
9	AKT1	AKT serine/threonine kinase 1	9.22E + 13	4.00	173.00	1,687.52	191.50
9	MMP2	Matrix metallopeptidase 2	9.22E + 13	5.00	99.00	220.08	154.50
9	SRC	SRC proto-oncogene, non-receptor tyrosine kinase	9.22E + 13	5.00	134.00	766.75	172.00
Rosuvastatin							
1	ALB^*^	Albumin	9.22E + 13	44.00	93.00	1,177.20	105.08
2	AKT1^*^	AKT serine/threonine kinase 1	9.22E + 13	11.00	89.00	878.05	103.08
3	TNF^*^	Tumor necrosis factor	9.22E + 13	8.00	97.00	1,270.34	106.92
4	IL1B^*^	Interleukin 1 beta	9.22E + 13	6.00	79.00	557.01	97.92
5	SELE	Selectin E	1.82E + 13	5.00	36.00	94.79	76.83
6	CAT	Catalase	9.22E + 13	4.00	48.00	194.11	82.58
6	PTGIS	Prostaglandin I2 synthase	279	5.00	12.00	400.50	61.67
8	MDM2	MDM2 proto-oncogene	9.22E + 13	2.00	33.00	50.64	74.42
8	ESR1	Estrogen receptor 1	9.22E + 13	3.00	57.00	260.82	86.92
8	CASP3^*^	Caspase 3	9.22E + 13	3.00	65.00	379.67	90.92
8	PECAM1	Platelet and endothelial cell adhesion molecule 1	9.22E + 13	1.00	50.00	129.10	83.42
8	MMP9^*^	Matrix metallopeptidase 9	9.22E + 13	5.00	77.00	474.21	96.92
Sirolimus + Rosuvastatin							
1	ALB^*^	Albumin	9.22E + 13	40.00	79.00	992.34	87.00
2	AKT1^*^	AKT serine/threonine kinase 1	9.22E + 13	11.00	75.00	629.42	85.00
3	TNF^*^	Tumor necrosis factor	9.22E + 13	5.00	82.00	952.30	88.50
3	ESR1	Estrogen receptor 1	9.22E + 13	5.00	49.00	194.33	71.83
5	MMP9^*^	Matrix metallopeptidase 9	9.22E + 13	3.00	66.00	404.38	80.50
5	IL1B	Interleukin 1 beta	9.22E + 13	7.00	67.00	418.99	81.00
7	IGF1	Insulin-like growth factor 1	9.22E + 13	3.00	54.00	190.52	74.50
7	CAT	Catalase	9.22E + 13	3.00	38.00	110.59	66.50
7	CASP3^*^	Caspase 3	9.22E + 13	3.00	58.00	322.68	76.50
10	SRC^*^	SRC proto-oncogene, non-receptor tyrosine kinase	9.22E + 13	2.00	57.00	227.32	76.00
10	PPARA	Peroxisome proliferator-activated receptor alpha	2.99E + 12	1.00	31.00	85.01	63.00
10	HSP90AA1^*^	Heat shock protein 90 alpha family class A member 1	9.22E + 13	2.00	48.00	162.96	71.50
10	HMOX1	Heme oxygenase 1	3.46E + 12	2.00	34.00	228.22	64.50
10	MDM2	MDM2 proto-oncogene	9.22E + 13	2.00	31.00	37.13	62.83
10	SERPINA1	Serpin family A member 1	1,670,018	2.00	22.00	70.77	58.33
10	MMP2	Matrix metallopeptidase 2	7.18E + 13	2.00	47.00	127.75	71.00

*MCC* maximal clique centrality, *DC* degree centrality, *BC* betweenness centrality, *CC* closeness centrality

^*^Asterisks indicate genes with overlapping hub-bottlenecks

#### Sirolimus and CABG PPI network analysis

The network complex between sirolimus and CABG comprised 211 nodes and 6086 edges. We identified the top 10 hub genes as beta-actin, *JUN*, *STAT3*, vascular endothelial growth factor A, hypoxia-inducible factor 1 subunit alpha, *IL6*, albumin (*ALB*), caspase 3 (*CASP3*), catenin beta 1, and tumor protein 53 (*TP53*). The top 11 bottleneck genes were *TP53*, *IL6*, *STAT3*, vascular cell adhesion molecule 1, *IL1B*, *MAPK14*, estrogen receptor 1 (*ESR1*), *ALB*, *AKT1*, *MMP2*, and SRC proto-oncogene (*SRC*) (Fig. 6A).

#### Rosuvastatin and CABG PPI network analysis

The network complex between rosuvastatin and CABG included 119 nodes and 1695 edges. We identified the top 10 hub genes as *TNF*, *ALB*, *MMP9*, *AKT1*, insulin-like growth factor 1 (*IGF1*), epidermal growth factor receptor (*EGFR*), *CASP3*, *SRC*, *IL1B*, and HRas proto-oncogene (*HRAS*). The top 12 bottleneck genes were *ALB*, *AKT1*, *TNF*, *IL1b*, selectin E, catalase (*CAT*), prostaglandin I2 synthase, MDM2 proto-oncogene (*MDM2*), *ESR1*, *CASP3*, platelet and endothelial cell adhesion molecule 1, and *MMP9* (Fig. 6B).

#### Combined sirolimus, rosuvastatin, and CABG PPI network analysis

The network complex involving sirolimus, rosuvastatin, and CABG comprised 96 nodes and 1,218 edges. The top 10 hub genes included *TNF*, *ALB*, *MMP9*, *AKT1*, *SRC*, *CASP3*, *EGFR*, *HRAS*, heat shock protein 90 alpha family class A member 1 (*HSP90AA1*), and *MAPK8*. The top 16 bottleneck genes were *ALB*, *AKT1*, *TNF*, *ESR1*, *MMP9*, *IL1B*, *IGF1*, *CAT*, *CASP3*, *SRC*, peroxisome proliferator-activated receptor alpha, *HSP90AA1*, heme oxygenase 1, *MDM2*, serpin family A member 1, and *MMP2* (Fig. 6C). Taken together, these data help predict the potential mechanisms by identifying key genes associated with sirolimus and rosuvastatin during NI formation and those influenced when both drugs are combined.

### Evaluating the efficacy of sirolimus and rosuvastatin in preventing NIH in a rabbit model

To investigate the therapeutic effects of the combined use of sirolimus and rosuvastatin, we developed a localized perivascular drug delivery device that contained both drugs. This device was designed based on the results of a network pharmacology analysis. The device was strategically designed to release sirolimus initially, followed by a sustained release of rosuvastatin (Additional file 1: Fig. S1A). Through in vitro testing, we confirmed the device's local drug delivery capability (Additional file 1: Fig. S1B). The structure and surface of the device were examined using optical microscopy and SEM to verify both the 2 × 4 format of the microneedles in the silk wrap and the presence or absence of the drug application (Additional file 1: Fig. S1C and D).

**Fig. 7:**
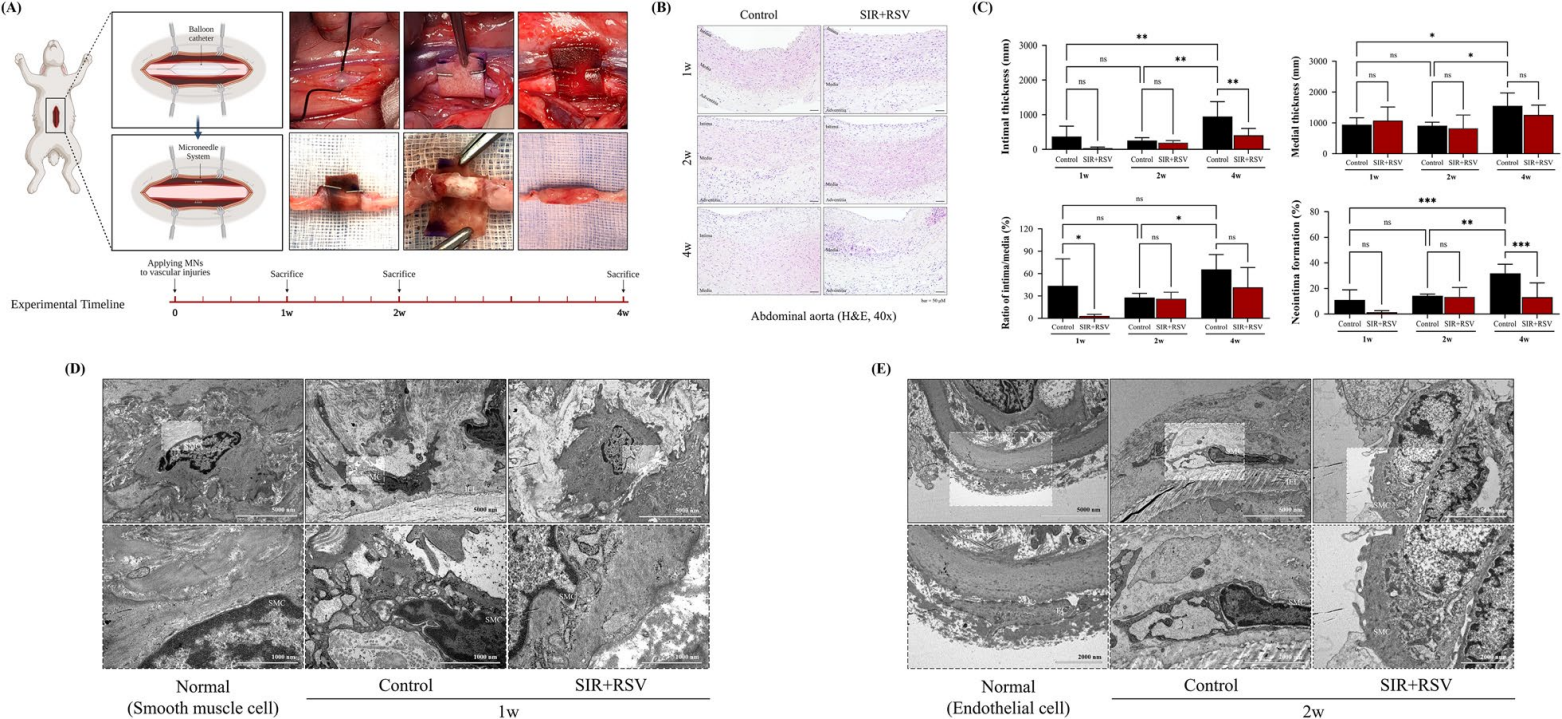
Sirolimus and rosuvastatin co-treatment alleviate balloon injury-induced intimal hyperplasia in an experimental model. **A** Schematic illustration of the injury and application protocol. The rabbit abdominal aortas were injured using a balloon catheter and treated using a localized perivascular drug delivery device containing either a control or SIR + RSV. Blood vessels were harvested after 1, 2, and 4 weeks. The figure was created using BioRender. **B** Histological changes in the aortas were observed using hematoxylin and eosin staining after control or SIR + RSV treatment (scale bars = 50 μm). **C** Quantification of intimal and medial thickness, the ratio of intima/media, and neointima formation. **C**–**E** Transmission electron microscopy images of vascular smooth muscle cells in the treated aorta after 1 and 2 weeks and in the native aorta (Normal). Statistical significance was determined using Fisher’s least significant difference test (*p < 0.05, **p < 0.01, ***p < 0.001). *SIR + RSV* combination of sirolimus and rosuvastatin

Figure 7A illustrates the in vivo validation experiment design, outlining the injury and application protocol. We subjected rabbit abdominal aortas to balloon catheter-induced injury, and then applied either a control device (no drugs) or the localized perivascular drug delivery device containing SIR + RSV. No toxicity or complications were observed in any of the groups (Additional file 1: Table S1 and Fig. S2).

Histological changes were observed in the H&E-stained aortic sections at 1, 2, and 4 weeks (Fig. 7B and C). In the control group, there was a significant increase in both intimal and medial thickness, as well as NI formation, over time. The intima-to-media ratio was significantly lower in the SIR + RSV group at 1 week compared to the control group (3.24 ± 2.08 and 43.60 ± 36.14, respectively). Additionally, after 4 weeks, the SIR + RSV group showed significant reductions in intimal thickness and NI formation compared to the control group (Control: 947.64 ± 429.14 mm and 31.73 ± 7.16%; SIR + RSV: 406.74 ± 196.18 mm and 13.15 ± 11.18%, respectively).

Based on these observations, TEM images at 1 and 2 weeks showed a reduction in the neointimal layer formation in the SIR + RSV group compared to the control group (Fig. 7D and E; Additional file 1: Fig. S3). At 1 week, the VSMCs in the control group displayed deformations, whereas the SIR + RSV group did not exhibit such alterations compared to the normal group. At 2 weeks, VSMCs had migrated within the neointimal layer in the control group. However, the VSMCs underwent cell death in the SIR + RSV group. These findings suggest that the combination of sirolimus and rosuvastatin effectively prevented NIH by inhibiting VSMC proliferation and migration.

### Inhibition of pro-inflammatory factor production by sirolimus and rosuvastatin in the acute stage of NIH progression

Our network pharmacology analysis identified potential interactions between sirolimus, rosuvastatin, and the regulation of inflammatory responses associated with CABG-related disease targets. While sirolimus appeared to influence the acute stage of NIH progression via IL-6/STAT3 signaling, rosuvastatin was found to be associated with the chronic stage, primarily via TNF-α-dependent MMP9 signaling.

**Fig. 8:**
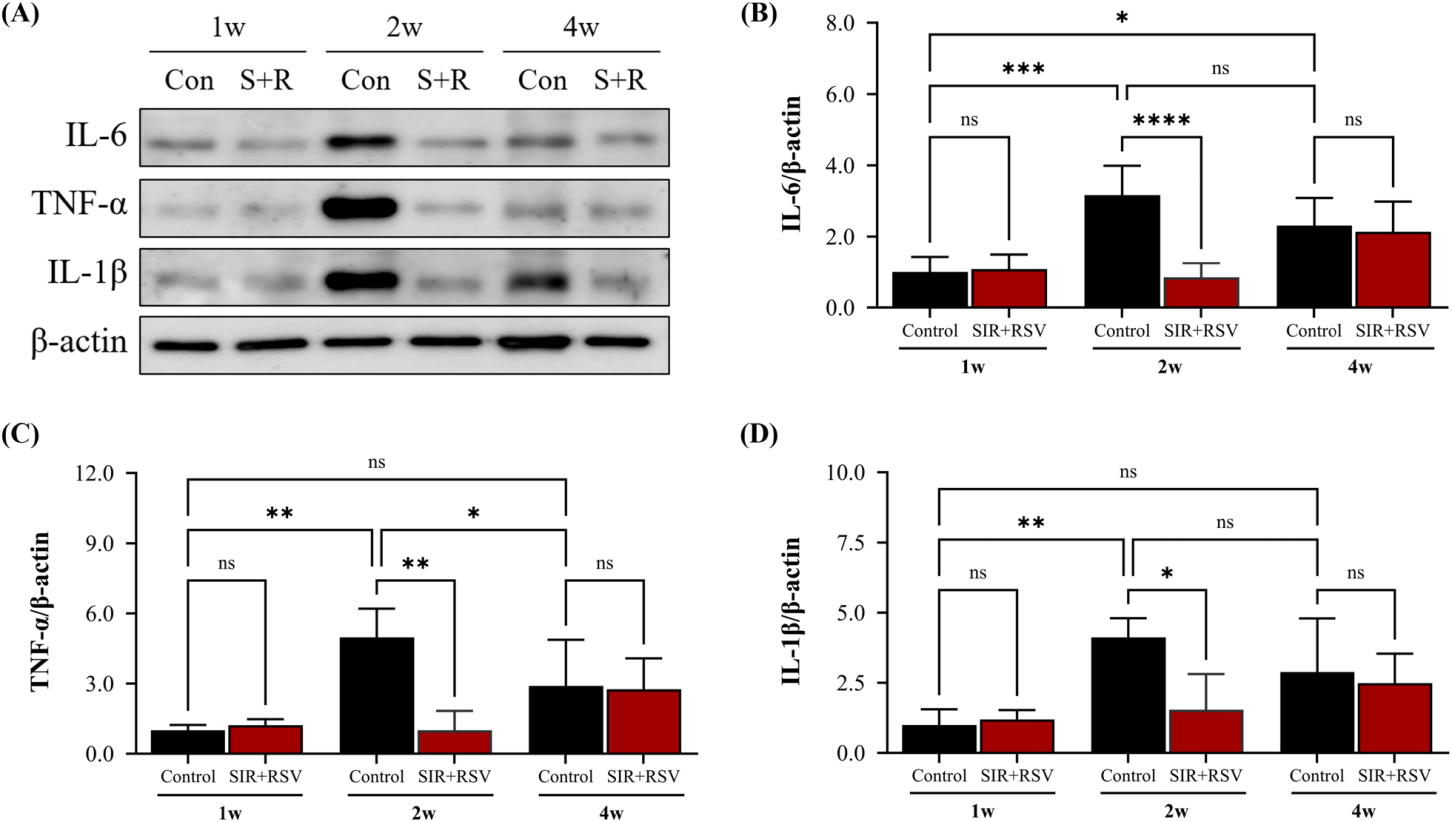
Sirolimus and rosuvastatin inhibit pro-inflammatory cytokine production in the acute stage. **A**–**D** Protein levels of inflammatory cytokines IL-6, TNF-α, and IL-1β were detected via western blot analysis. Data are presented as the mean ± standard error of mean. Statistical significance was determined using Fisher’s least significant difference test (* p < 0.05, ** p < 0.01, ***p < 0.001). *SIR + RSV* combination of sirolimus and rosuvastatin, *IL-6* interleukin 6, *TNF-α* tumor necrosis factor alpha, *IL-1β* interleukin 1 beta

To validate these findings, we assessed the changes in the inflammatory response in balloon-injured rabbit models treated with a device containing sirolimus and rosuvastatin. At 2 weeks, the control group showed a significant increase in the expression of pro-inflammatory cytokines, such as IL-6, TNF-α, and IL-1β (Fig. 8). In contrast, in comparison to the control group, the SIR + RSV group demonstrated significant reductions in the expression of IL-6, TNF-α, and IL-1β at 2 weeks (4.37-, 4.95-, and 2.67-fold decreases, respectively). Notably, sirolimus and rosuvastatin effectively reduced pro-inflammatory cytokine expression at 2 weeks (acute stage), suggesting that these drugs prevent NIH by negatively regulating cytokine production.

### Attenuation of STAT3 activation by sirolimus and rosuvastatin in the chronic stage of NIH progression

**Fig. 9:**
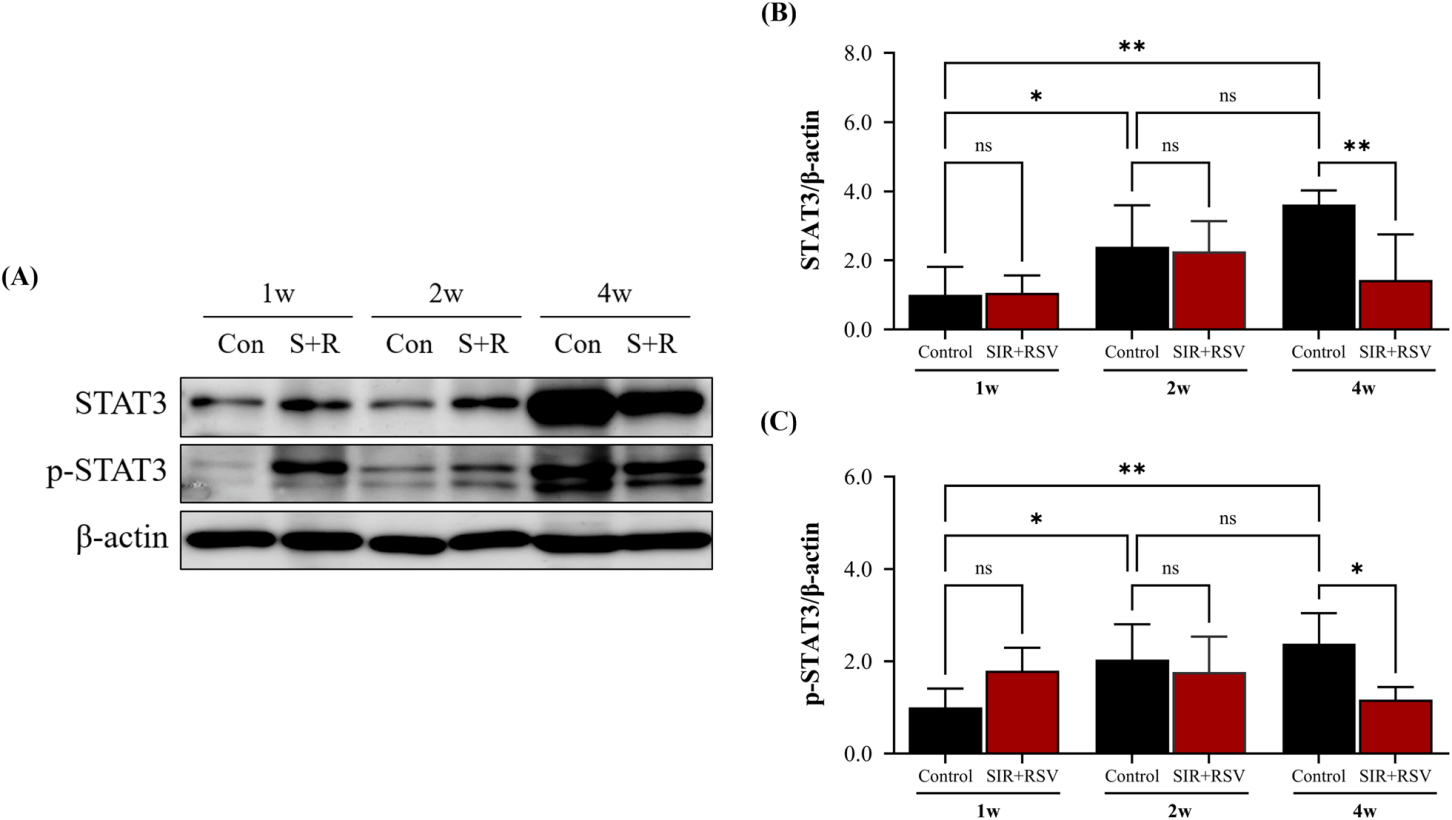
Sirolimus and rosuvastatin attenuate STAT3 phosphorylation in the chronic stage. **A**–**C** Protein levels of STAT3 and p-STAT3 were determined via western blot analysis. Data are presented as the mean ± standard error of mean. Statistical significance was determined using Fisher’s least significant difference test (*p < 0.05, **p < 0.01, ***p < 0.001). *SIR + RSV* combination of sirolimus and rosuvastatin, *STAT3* signal transducer and activator of transcription 3, *p-STAT3* phosphorylated STAT3

Following the observed impacts of sirolimus and rosuvastatin that affected pro-inflammatory cytokine production, we next investigated the role of *IL-6* and *STAT3*, which were the hub genes associated with sirolimus and CABG, as identified in our PPI analysis. Previous studies have confirmed that the IL-6/STAT3 signaling pathway exhibits abnormal overactivation in chronic inflammatory conditions [44]. STAT3 activity is of paramount importance in a multitude of biological processes, including cell proliferation, apoptosis, differentiation, and VSMC phenotype switching [45, 46]. Accordingly, we investigated the alterations in the protein levels of STAT3 and p-STAT3 (Fig. 9). Western blot analysis demonstrated a time-dependent increase in the expression of STAT3 and p-STAT3. In contrast, the expression levels were significantly decreased in the SIR + RSV group compared to those in the control group at 4 weeks, with reductions of 2.53-fold and 2.04-fold, respectively. Taken together with our IL-6 expression results, we suggest that sirolimus and rosuvastatin effectively inhibit cell proliferation and survival during the chronic stage by attenuating STAT3 activation via IL-6 inhibition in the acute stage.

### *Modulation of MMP expression by sirolimus and rosuvastatin *via* the Akt/mTOR/NF-kB signaling pathway during the chronic stage of NIH progression*

**Fig. 10:**
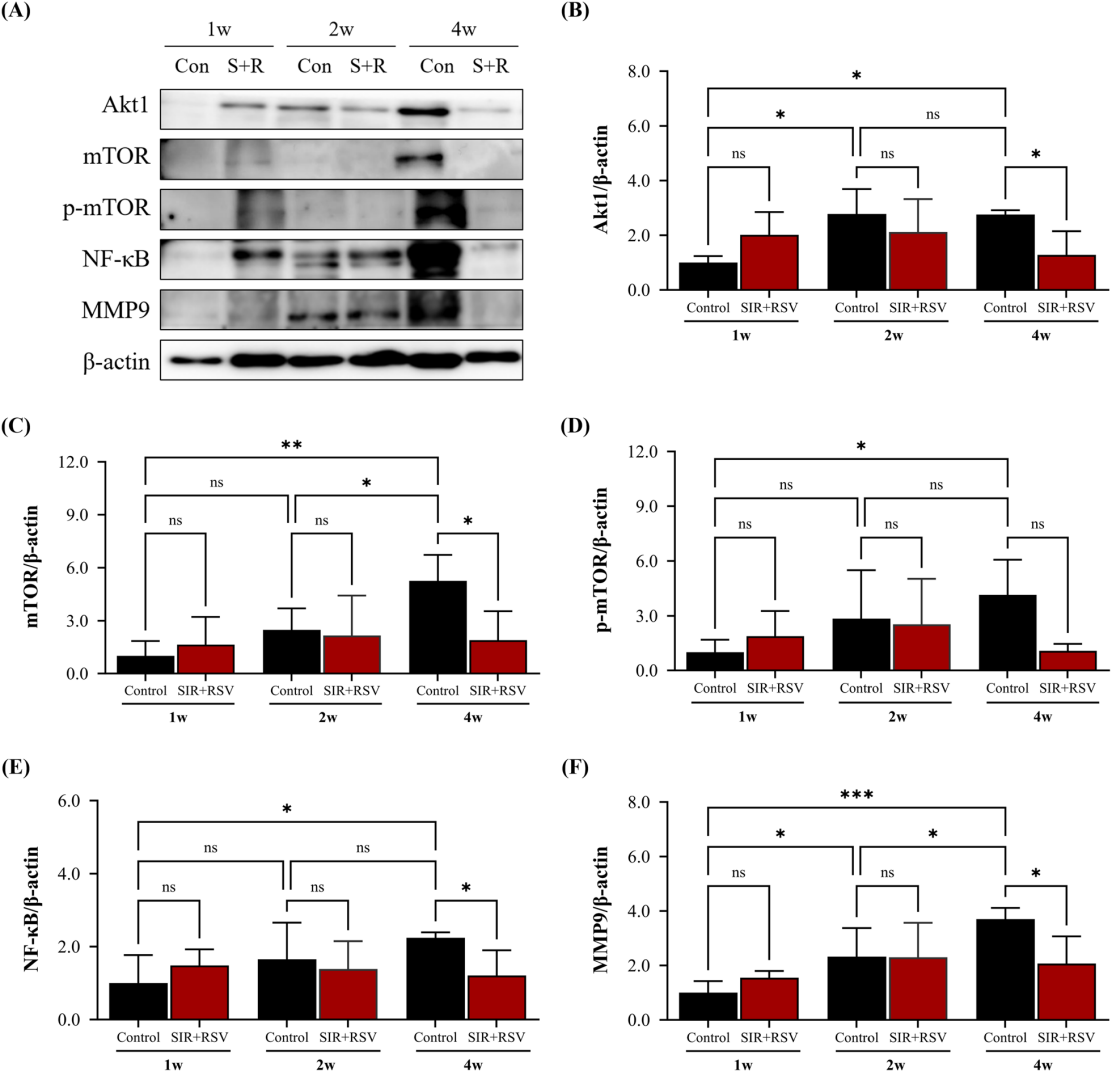
Sirolimus and rosuvastatin regulate MMP expression in the chronic stages via Akt/mTOR/NF-kB signaling. **A**–**F** Protein levels of Akt1, mTOR, p-mTOR, NF-κB, and MMP9 were detected via western blot analysis. Data are presented as the mean ± standard error of mean. Statistical significance was determined using Fisher’s least significant difference test (*p < 0.05, **p < 0.01, ***p < 0.001). *SIR + RSV* combination of sirolimus and rosuvastatin, *Akt* a protein cloned from the v-akt oncogene of retrovirus AKT8, *mTOR* mammalian target of rapamycin, *p-mTOR* phosphorylated mTOR, *NF-κB* nuclear factor kappa-light-chain-enhancer of activated B cells, *MMP* matrix metalloproteinase

To further elucidate the pro-inflammatory cytokine-related mechanisms, we investigated the roles of TNF, AKT1, and MMP9— key genes from our PPI analysis— in association with sirolimus, rosuvastatin, and CABG. TNF-α, a key modulator of VSMC phenotypic changes during NIH progression, activates the AKT/mTOR pathway and augments MMP9 expression via NF-κB activation in VSMCs [47, 48]. Therefore, we investigated the impact of combining sirolimus and rosuvastatin on factors associated with the AKT/mTOR/NF-κB signaling pathway (Fig. 10). Compared to the control group, the expression levels of Akt1, mTOR, NF-κB, and MMP9 in the SIR + RSV group were significantly reduced at 4 weeks, showing 2.15-fold, 2.76-fold, 1.85-fold, and 1.79-fold reductions, respectively. Moreover, p-mTOR expression showed a decreasing trend in the SIR + RSV group (3.83-fold decrease; p = 0.0594). These findings indicate that the combination of sirolimus and rosuvastatin can effectively inhibit VSMC proliferation and prevent phenotypic alterations in the chronic stage of NIH, primarily by modulating MMP expression via the TNF-α-dependent Akt/mTOR/NF-κB signaling pathway.

## Discussion

Neointimal hyperplasia (NIH) of graft and anastomotic sites, which profoundly impacts the long-term patency and outcomes of vascular conduits after CABG, primarily unfolds via three significant mechanisms: (1) hyper-acute stage involving endothelial damage and platelet accumulation; (2) acute stage involving endothelial dysfunction and inflammation; and (3) chronic stage involving VSMC proliferation and migration, ECM synthesis, and NI formation [11, 49, 50]. Sirolimus and rosuvastatin are recognized for their effectiveness in preventing NIH [20, 51] and represent potential pharmacological agents to improve long-term patency following CABG. However, the efficacy of this combination remains unclear. In the present study, a network pharmacology analysis was conducted, followed by in vivo validation, to investigate the potential of combining sirolimus and rosuvastatin for the prevention of NIH (Fig. 1).

Network pharmacology can predict targets associated with the mechanisms of drugs and diseases as well as interactions between different drugs [52, 53]. We identified potential therapeutic targets of sirolimus and rosuvastatin in the context of NIH (Fig. 2). Functional enrichment analysis revealed a significant association between these targets and biological processes associated with the regulation of apoptosis, inflammatory response, as well as the PI3K-Akt and TNF signaling pathways (Figs. 3, 4, 5). PPI analysis revealed several key overlapping hub-bottleneck genes integral to NIH prevention: (1) sirolimus: *STAT3*, *IL6*, *ALB*, and *TP53*; (2) rosuvastatin: *TNF*, *ALB*, *MMP9*, *AKT1*, *CASP3*, and *IL1B*; and (3) sirolimus and rosuvastatin: *TNF*, *ALB*, *MMP9*, *AKT1*, *SRC*, *CASP3*, and *HSP90AA1* (Fig. 6 and Table 1). From a network pharmacology perspective, each drug interacts with specific targets during different stages of NIH progression. Specifically, sirolimus primarily targets the acute stage by regulating the IL-6/STAT3 signaling pathway, whereas rosuvastatin is more associated with the chronic stage and affects the TNF-α-dependent MMP9 signaling pathway. Notably, both drugs influenced the PI3K-Akt signaling pathway as well as inflammation-related pathways, suggesting the possibility of synergistic effects. Based on these findings, we hypothesized that administering sirolimus and rosuvastatin at their respective stages of NIH may enhance their synergistic effects in preventing NIH.

Although the synergistic effects of sirolimus and rosuvastatin offer potential benefits, the possibility of systemic side effects necessitates an alternative delivery method [54]. Local drug delivery has been widely studied owing to its capability to sustain an effective drug concentration at the target site while significantly minimizing systemic toxicity [55]. To mitigate these side effects, we used a local perivascular drug delivery device that was developed in our previous investigation [13]. The device was designed to contain both drugs and possess specific release mechanisms based on the requirement for each drug: an early release of sirolimus and a sustained release of rosuvastatin (Additional file 1: Fig. S1). In our study, neither the control group nor the SIR + RSV group demonstrated any signs of toxicity or complications, such as hepatotoxicity, nephrotoxicity, or skeletal muscle toxicity, throughout the duration of the procedures (Additional file 1: Table S1 and Fig. S2). Additionally, the utilization of the delivery device containing sirolimus and rosuvastatin resulted in a significant reduction in intimal thickness and NI formation in comparison to that in the control group at 4 weeks (Fig. 7). The findings of this study suggest that the localized perivascular delivery of sirolimus and rosuvastatin can successfully inhibit NI formation without causing any side effects.

Next, to further investigate our hypothesis, we evaluated the mechanisms of NIH prevention. Inflammation plays a crucial role in NIH progression and is closely linked to tissue healing following surgical trauma. In the acute stage of NIH, leukocytes penetrate the vascular wall, triggering the secretion of pro-inflammatory cytokines and growth factors. These factors create a stimulatory environment conducive to the proliferation and migration of VSMCs [11]. In our study, the control group showed a significant increase in the levels of pro-inflammatory cytokines IL-6, TNF-α, and IL-1β in the acute stage. In contrast, the levels of cytokines were significantly decreased in the group co-treated with sirolimus and rosuvastatin. These results emphasize the important role of inflammation in the progression of NIH, and suggest that a combined therapeutic strategy can effectively prevent NIH progression by modulating inflammatory cytokine secretion.

Based on the network pharmacology analysis results, we could describe the associations between pro-inflammatory cytokines and key proteins, such as STAT3, AKT, and MMP9, in the context of sirolimus and rosuvastatin combination therapy for NIH prevention. In addition, western blot analysis confirmed that the combination drug treatment in the chronic phase reduced NF-κB and STAT3 activation and decreased MMP9 expression via the Akt/mTOR/NF-κB signaling pathway. Taken together, our data suggest that sirolimus and rosuvastatin exert their therapeutic effects by inhibiting pro-inflammatory cytokine production during the acute phase of NIH progression and modulating the Akt/mTOR/NF-κB/STAT3 signaling pathway during the chronic phase. These findings support the notion that a multi-targeting strategy utilizing these two drugs can effectively prevent NIH progression, including VSMC proliferation and migration as well as vascular remodeling.

## Limitations

The majority of our findings were obtained using aorta tissues and are not always directly applicable to other blood vessels. To comprehensively validate our findings, future studies should include vascular grafts and anastomosis sites, such as the coronary artery, internal mammary artery, radial artery, and saphenous vein. Additionally, the NI formation model used in our experiment, which involved endothelial cell denudation, did not fully reflect the conditions of CABG surgery. Future research should explore alternative drug delivery methods in the NIH model that simulate the CABG surgical technique. Despite these limitations, our findings have significant implications for preventing NIH of graft and anastomotic sites after CABG. Our findings indicate that strategically targeting specific stages of NIH using sirolimus and rosuvastatin can enhance their effectiveness in NIH prevention. Additionally, the innovative use of a localized perivascular drug delivery device addresses previous concerns regarding the systemic toxicity associated with oral administration of these drugs.

## Conclusions

**Fig. 11:**
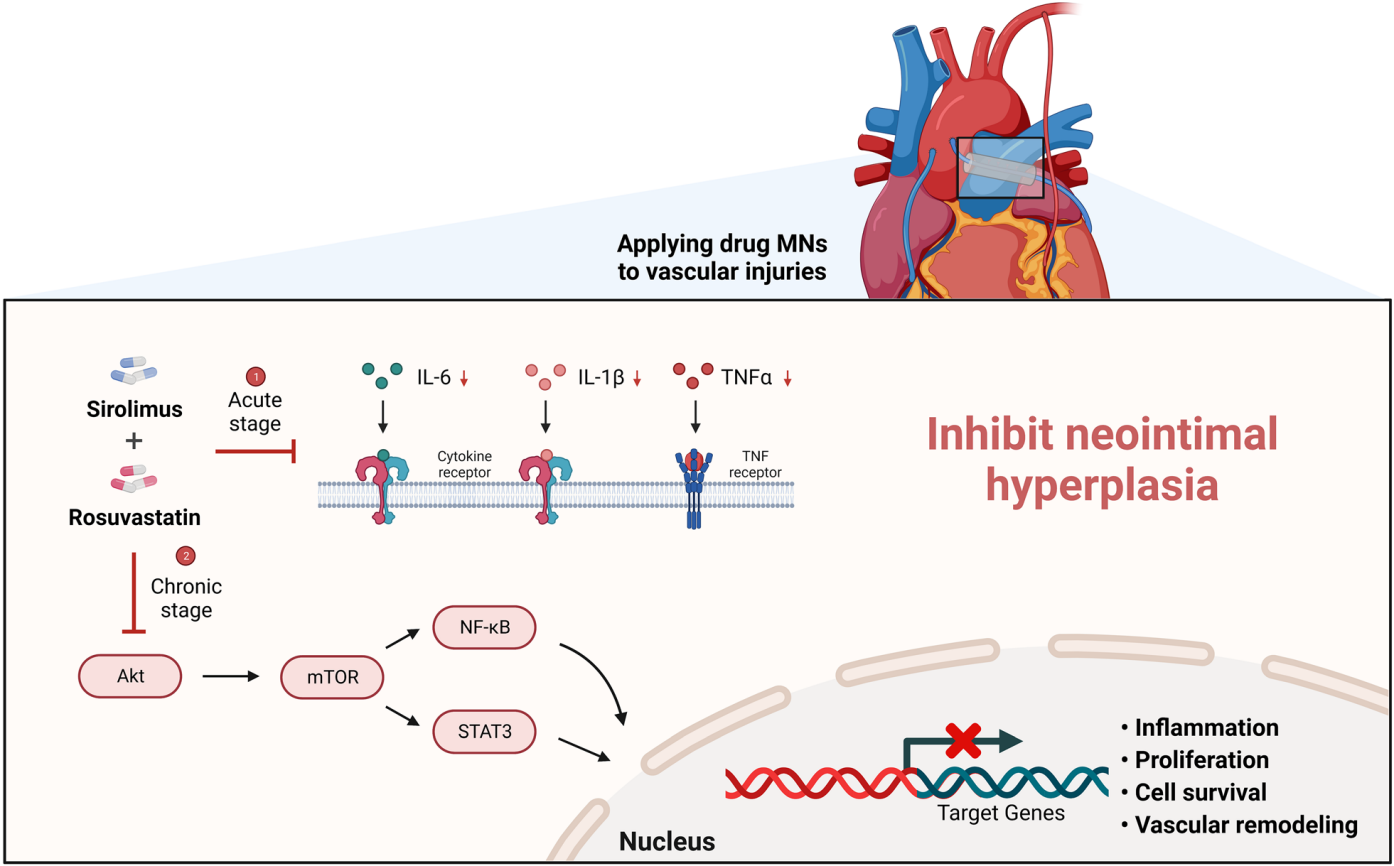
A schematic representation illustrating the role of sirolimus and rosuvastatin in preventing neointimal hyperplasia (created using BioRender). *IL-6* interleukin 6, *IL-1β* interleukin 1 beta, *TNF-α* tumor necrosis factor alpha, *Akt* a protein cloned from the v-akt oncogene of retrovirus AKT8, *mTOR* mammalian target of rapamycin, *STAT3* signal transducer and activator of transcription 3, *NF-κB* nuclear factor kappa-light-chain-enhancer of activated B cells

Sirolimus and rosuvastatin exert therapeutic effects against NIH progression by inhibiting the production of pro-inflammatory cytokines in the acute stage and modulating the Akt/mTOR/NF-κB/STAT3 signaling pathway in the chronic stage. These mechanisms primarily function to inhibit the proliferation, migration, and vascular remodeling of VSMCs, consequently mitigating the progression of NIH (Fig. 11). Our study highlights the potential therapeutic strategies for preventing NI formation of graft and anastomotic sites after CABG, particularly, the potential benefits of sirolimus and rosuvastatin combination treatment.

### Supplementary Information

**Additional file 1: Table S1. **Change in body weight over time in both groups. **Table S2.** Top 10 biological process enrichment analysis of overlapping targets of coronary artery bypass graft and sirolimus involved in neointimal hyperplasia. **Table S3.** Top 10 cellular component enrichment analysis of overlapping targets of coronary artery bypass graft and sirolimus involved in neointimal hyperplasia. **Table S4.** Top 10 molecular function enrichment analysis of overlapping targets of coronary artery bypass graft and sirolimus involved in neointimal hyperplasia. **Table S5.** Top 10 KEGG pathway enrichment analysis of overlapping targets of coronary artery bypass graft and sirolimus involved in neointimal hyperplasia. **Table S6.** Top 10 biological process enrichment analysis of overlapping targets of coronary artery bypass graft and rosuvastatin involved in neointimal hyperplasia. **Table S7.** Top 10 cellular component enrichment analysis of overlapping targets of coronary artery bypass graft and rosuvastatin involved in neointimal hyperplasia. **Table S8.** Top 10 molecular function enrichment analysis of overlapping targets of coronary artery bypass graft and rosuvastatin involved in neointimal hyperplasia. **Table S9.** Top 10 KEGG pathway enrichment analysis of overlapping targets of coronary artery bypass graft and rosuvastatin involved in neointimal hyperplasia. **Table S10.** Top 10 biological process enrichment analysis of overlapping targets of coronary artery bypass graft and both drugs involved in neointimal hyperplasia. **Table S11.** Top 10 cellular component enrichment analysis of overlapping targets of coronary artery bypass graft and both drugs involved in neointimal hyperplasia. **Table S12.** Top 10 molecular function enrichment analysis of overlapping targets of coronary artery bypass graft and both drugs involved in neointimal hyperplasia. **Table S13.** Top 10 KEGG pathway enrichment analysis of overlapping targets of coronary artery bypass graft and both drugs involved in neointimal hyperplasia. **Table S14.** Top 10 significant bottleneck genes ranked by CytoHubba. **Fig. S1.** Local drug delivery using a localized perivascular drug delivery device. **A** Diagram of the localized perivascular drug delivery device with sequential release of sirolimus and rosuvastatin. **B** In vitro test of the local drug delivery device. Fluorescence image of a spot inserted into an agarose gel after use of the localized perivascular drug delivery device. **C** Optical image and **D** scanning electron transmission image of the localized perivascular drug delivery device. **Fig. S2.** In vivo safety assessment of the localized perivascular drug delivery device. **A** Changes in body weight of the rabbits in each group during the 4-week experiment (n = 3–5/group). **B**–**D** Toxicity assessment of the localized perivascular drug delivery device via H&E staining in organs (liver, kidney, and skeletal muscle) of rabbits in the control and SIR + RSV groups. No significant pathological differences were observed between the two groups (scale bars = 100 μm). *H&E* hematoxylin and eosin, *SIR+RSV* combination of sirolimus and rosuvastatin. **Fig. S3.** Effect of the localized perivascular drug delivery device on cell morphology. **A** and **B** Transmission electron microscopy sections of rabbit abdominal aortas at both the hyper-acute stage (1 week) and the acute stage (2 weeks) after injury. The aortas were treated with a localized perivascular drug delivery device containing either no drug (control) or SIR+RSV. *SIR+RSV* combination of sirolimus and rosuvastatin. **Fig. S4.** Full-length western blot images. **A**–**C** Full-length western blot image for Figs. 8A, 9A, and 10A. SIR + RSV, combination of sirolimus and rosuvastatin. *IL-6* interleukin 6, *TNF-α* tumor necrosis factor alpha, *IL-1β* interleukin 1 beta, *STAT3* signal transducer and activator of transcription 3, *p-STAT3* phosphorylated STAT3, *Akt* a protein cloned from the v-akt oncogene of retrovirus AKT8, *mTOR* mammalian target of rapamycin, *p-mTOR* phosphorylated mTOR, *NF-κB* nuclear factor kappa-light-chain-enhancer of activated B cells, *MMP* matrix metalloproteinase.

## Data Availability

All data associated with this work are available in the table, figures, and additional file information.

## Abbreviations

Akt1: AKT serine/threonine kinase 1
ALB: Albumin
BPs: Biological processes
CABG: Coronary artery bypass graft
CAD: Coronary artery disease
CAT: Catalase
CCs: Cellular components
CTD: Comparative toxicogenomics database
ECM: Extracellular matrix
EGFR: Epidermal growth factor receptor
ESR1: Estrogen receptor 1
GO: Gene ontology
HRAS: Hras proto-oncogene
HSP90AA1: Heat shock protein 90 alpha family class A member 1
IGF1: Insulin-like growth factor 1
IL-6: Interleukin 6
KEGG: Kyoto encyclopedia of genes and genomes
MAPK: Mitogen-activated protein kinase
MCC: Maximal clique centrality
MDM2: MDM2 proto-oncogene
MFs: Molecular functions
MMP: Matrix metalloproteinase
mTOR: Mammalian target of rapamycin
NF-κB: Nuclear factor kappa-light-chain-enhancer of activated B cells
NI: Neointima
NIH: Neointimal hyperplasia
PI3K: Phosphoinositide 3-kinase
p-mTOR: Phosphorylated mammalian target of rapamycin
PPI: Protein–protein interaction
P-STAT3: Phosphorylated signal transducer and activator of transcription 3
SEM: Scanning electron microscopy
SIR + RSV: Sirolimus and rosuvastatin
SRC: SRC proto-oncogene
STAT3: Signal transducer and activator of transcription 3
TEM: Transmission electron microscopy
TNF-α: Tumor necrosis factor alpha
TP53: Tumor protein 53
VSMCs: Vascular smooth muscle cells
