# Supplementary material for: Prevention of neointimal hyperplasia after coronary artery bypass graft via local delivery of sirolimus and rosuvastatin: network pharmacology and in vivo validation
Source: J Transl Med. 2024 Feb 16;22:166. doi: 10.1186/s12967-024-04875-8 (PMC10874014; doi:10.1186/s12967-024-04875-8)
Supplement: Supplementary file 1 — Additional file 1: Table S1. Change in body weight over time in both groups. Table S2. Top 10 biological process enrichment analysis of overlapping targets of coronary artery bypass graft and sirolimus involved in neointimal hyperplasia. Table S3. Top 10 cellular component enrichment analysis of overlapping targets of coronary artery bypass graft and sirolimus involved in neointimal hyperplasia. Table S4. Top 10 molecular function enrichment analysis of overlapping targets of coronary artery bypass graft and sirolimus involved in neointimal hyperplasia. Table S5. Top 10 KEGG pathway enrichment analysis of overlapping targets of coronary artery bypass graft and sirolimus involved in neointimal hyperplasia. Table S6. Top 10 biological process enrichment analysis of overlapping targets of coronary artery bypass graft and rosuvastatin involved in neointimal hyperplasia. Table S7. Top 10 cellular component enrichment analysis of overlapping targets of coronary artery bypass graft and rosuvastatin involved in neointimal hyperplasia. Table S8. Top 10 molecular function enrichment analysis of overlapping targets of coronary artery bypass graft and rosuvastatin involved in neointimal hyperplasia. Table S9. Top 10 KEGG pathway enrichment analysis of overlapping targets of coronary artery bypass graft and rosuvastatin involved in neointimal hyperplasia. Table S10. Top 10 biological process enrichment analysis of overlapping targets of coronary artery bypass graft and both drugs involved in neointimal hyperplasia. Table S11. Top 10 cellular component enrichment analysis of overlapping targets of coronary artery bypass graft and both drugs involved in neointimal hyperplasia. Table S12. Top 10 molecular function enrichment analysis of overlapping targets of coronary artery bypass graft and both drugs involved in neointimal hyperplasia. Table S13. Top 10 KEGG pathway enrichment analysis of overlapping targets of coronary artery bypass graft and both drugs involved in neointimal hy [file 12967_2024_4875_MOESM1_ESM.docx]

Supporting Information

**Prevention of neointimal hyperplasia after coronary artery bypass graft via local delivery of sirolimus and rosuvastatin: network pharmacology and *in vivo* validation**

Ji-yeon Ryu^1,†^, Eui Hwa Jang^1,†^, JiYong Lee^2,3^, Jung-Hwan Kim^1^, Young-Nam Youn^1,*^

^1^Division of Cardiovascular Surgery, Department of Thoracic and Cardiovascular Surgery, Severance Cardiovascular Hospital, Yonsei University College of Medicine, Seoul 03722, South Korea

^2^ School of Mechanical Engineering, Yonsei University, Seoul 03722, South Korea

^3^ Department of Mechanical Engineering, University of Minnesota, Minneapolis, MN, 55455, USA

*** Contact information of corresponding author**

Professor Young-Nam Youn

E-mail: ynyoun@yuhs.ac

Telephone: +82-2 228 8487

^†^These authors contributed equally to this work.

**Table S1.** Change in body weight over time in both groups

| **Endpoint** | **Time (weeks)** | **Control** | **SIR+RSV^1)^** | **P-value** |
| --- | --- | --- | --- | --- |
| 1w | 0 (baseline) | 3.35 ± 0.35^2)^ | 3.54 ± 0.21 | 0.3942^3)^ |
|  | 1 | 3.20 ± 0.28 | 3.41 ± 0.25 | 0.3999 |
| 2w | 0 (baseline) | 3.40 ± 0.57 | 3.57 ± 0.12 | 0.6285 |
|  | 1 | 3.25 ± 0.49 | 3.43 ± 0.04 | 0.6593 |
|  | 2 | 3.30 ± 0.57 | 3.55 ± 0.07 | 0.5984 |
| 4w | 0 (baseline) | 3.68 ± 0.40 | 3.44 ± 0.32 | 0.3610 |
|  | 1 | 3.49 ± 0.44 | 3.30 ± 0.33 | 0.5447 |
|  | 2 | 3.69 ± 0.52 | 3.39 ± 0.33 | 0.3771 |
|  | 4 | 3.93 ± 0.55 | 3.53 ± 0.24 | 0.2323 |

^1)^ SIR+RSV, combination of sirolimus and rosuvastatin; ^2)^ Values are presented as mean ± SD (kg); ^3)^ Unpaired Student’s t-test was used for statistical analysis; no significant difference was found between the control and SIR+RSV groups.

**Table S2.** Top 10 biological process enrichment analysis of overlapping targets of coronary artery bypass graft and sirolimus involved in neointimal hyperplasia

| **Biological process** | **Genes** | **Adjusted p-value** |
| --- | --- | --- |
| Negative regulation of apoptotic process | RB1, FHL2, TNF, MPO, IGF1R, AKT2, MYC, CASP3, KDR, AKT1, CTSG, IL10, PRKCD, F2R, MIF, SIRT1, MMP9, PIK3CA, CAT, BIRC5, TP53, SQSTM1, HDAC2, PRKAA1, SRC, PIK3R1, EGFR, RELA, MAPK8, MAPK1, PPARGC1A, MCL1, MAPK3, XBP1, HSPA5, IGF1, PTK2, IL2, NFKB1, VEGFA, SOD1, IL4, NFKBIA, IL6, RPS6KB1, ALB, CDK1, FAS, CTNNB1, TEK, BCL2L1 | 1.80385E-33 |
| Cellular response to oxygen-containing compound | CXCL8, NCF1, IRS1, SERPINE1, PDGFB, ACTB, EGFR, RELA, MAPK8, CASP1, AKT1, NLRP3, MAPK1, CTSG, TGM2, MAPK3, IL10, P2RY12, XBP1, JUN, DAPK1, PRKCD, MMP3, NR1H3, MAPK14, ESR1, MMP9, RHOA, NFKB1, MTOR, CXCL10, IL6, CREB1, IL1B, CDK2, VIM | 3.09319E-24 |
| Regulation of inflammatory response | RB1, SERPINE1, TNF, RELA, ALOX5, CASP1, NLRP3, ELANE, IL10, CMA1, PRKCD, MMP3, IL18, NR1H3, SELE, MMP9, AGT, IL2, IL4, NFKBIA, ACE2, IL6, IFNG, IL1B, PPARG, TEK, PPARA, S100A9 | 4.553E-21 |
| Cellular response to oxidative stress | JUN, PRKAA1, NCF1, NCF2, DAPK1, PRKCD, CYBA, FOXO3, HIF1A, EIF2S1, MMP9, EGFR, SOD1, MAPK8, CAT, AKT1, HMOX1, MAPK1, TP53, PPARGC1A, NFE2L2, MAPK3 | 5.81443E-19 |
| Vascular endothelial growth factor receptor signaling pathway | HSP90AA1, NCF1, NCF2, SRC, CYBA, PIK3CB, PIK3R1,  MAPK14, RHOA, PGF, PTK2, VEGFA, CDC42, PIK3CA, KDR, RAC1 | 6.01571E-16 |
| Extracellular matrix organization | SERPINE1, PDGFB, CTSS, ICAM1, TTR, CTSK, KDR, SPP1, CTSG, ELANE, POSTN, TGFB2, TGFB1, VCAM1, MMP7, MMP1, CMA1, MMP2, MMP3, FN1, MMP9, MMP12, MMP14, VCAN, COL1A2, ITGA6 | 8.47536E-15 |
| Regulation of blood vessel endothelial cell migration | TGFB1, NOS3, KDR, PDGFB, AKT1, PPARG, MAPK14, TNF, SIRT1, HIF1A, VEGFA, FGFR1 | 1.39621E-11 |
| Regulation of vascular associated smooth muscle cell proliferation | IL10, CDKN1A, JUN, CDKN1B, MMP2, PDGFB, PTEN, PPARG, IGF1, MMP9 | 1.08492E-10 |
| Platelet degranulation | TGFB2, TGFB1, SERPINA1, EGF, SERPINE1, PDGFB, IGF2, FN1,IGF1, VEGFA, SOD1, SELP, ALB, TIMP3, CD36 | 1.23604E-10 |
| Regulation of nitric-oxide synthase activity | HSP90AA1, CAV3, NOS3, IL1B, AKT1, PIK3CB, TNF, HIF1A, EGFR, INS | 1.81453E-10 |

**Table S3.** Top 10 cellular component enrichment analysis of overlapping targets of coronary artery bypass graft and sirolimus involved in neointimal hyperplasia

| **Cellular component** | **Genes** | **Adjusted p-value** |
| --- | --- | --- |
| Secretory granule lumen | SERPINA1, SERPINE1, PDGFB, MPO, INS, TTR, ALOX5, TIMP3, MAPK1, CTSG, ELANE, TGFB2, HSP90AA1, TGFB1, EGF, PRKCD, FN1, IGF2, MIF, IGF1, MAPK14, NFKB1, VEGFA, PKM, ALB, CAT, LCN2, S100A9 | 9.65342E-16 |
| Collagen-containing extracellular matrix | SERPINA1, SERPINE1, PDGFB, CTSS, ICAM1, LGALS3, NPPA, TIMP3, CTSG, CCN2, ELANE, TGM2, POSTN, TGFB2, TGFB1, CMA1, MMP2, FN1, F2, F3, MMP9, AGT, VCAN, COL1A2, PKM, S100A9, FGFR2 | 4.95029E-13 |
| Platelet alpha granule | TGFB2, SERPINA1, TGFB1, EGF, SERPINE1, PDGFB, FN1, IGF2, IGF1, VEGFA, SELP, ALB, CD36 | 4.29879E-10 |
| Endoplasmic reticulum lumen | SERPINA1, F10, HSPA5, PDGFB, FN1, APOA2, F2, CP, INS, ACE2, IL6, VCAN, COL1A2, BGLAP, ALB, SPP1, MAPK1, IL12A, PNPLA2, MAPK3 | 8.53162E-10 |
| Platelet alpha granule lumen | TGFB2, SERPINA1, TGFB1, EGF, SERPINE1, ALB, PDGFB, FN1, IGF2, IGF1, VEGFA | 2.56541E-09 |
| Focal adhesion | MME, CAV3, HSPA5, SRC, FHL2, ACTB, PTK2, RHOA, EGFR, ICAM1, CDC42, MMP14, PTPRC, PLAU, CAT, CTNNB1, MAPK1, ITGA6, RAC1, VIM, TGM2, MAPK3 | 3.32203E-09 |
| Cell-substrate junction | MME, CAV3, HSPA5, SRC, FHL2, ACTB, PTK2, RHOA, EGFR, ICAM1, CDC42, MMP14, PTPRC, PLAU, CAT, CTNNB1, MAPK1, ITGA6, RAC1, VIM, TGM2, MAPK3 | 4.20519E-09 |
| Caveola | CAV3, IRS1, SRC, NOS3, F2R, MAPK1, CD36, SELE, MAPK3 | 2.22182E-07 |
| Membrane raft | ABCA1, CAV3, MME, SRC, TNF, SELE, EGFR, ACE2, PTPRC, KDR, FAS, CD36, TEK | 3.12926E-07 |
| Cyclin-dependent protein kinase holoenzyme complex | CDKN1A, CCND1, CCNE2, CCNE1, CDK4, CDK2, CDK1 | 3.23855E-07 |

**Table S4.** Top 10 molecular function enrichment analysis of overlapping targets of coronary artery bypass graft and sirolimus involved in neointimal hyperplasia

| **Molecular function** | **Genes** | **Adjusted p-value** |
| --- | --- | --- |
| Receptor ligand activity | SEMA3A, PDGFB, FGF1, TNF, INS, NPPB, LGALS3, NPPA,  IL12A, IL10, EDN1, TGFB2, TGFB1, EGF, IGF2, IL18, MIF, IGF1, F2, AGT, PGF, IL2, VEGFA, IL4, CXCL10, BMP2, IL6, IFNG, IL1B | 8.17294E-17 |
| Endopeptidase activity | C1S, CTSS, CASP9, CASP7, CASP8, PLAU, CTSK, CASP3, CASP1, CTSG, ELANE, MMP7, MME, F10, MMP1, CMA1, MMP2, MMP3, F2, F3, MMP9, MMP12, ACE2, MMP14, REN | 8.7075E-13 |
| Kinase binding | RB1, BECN1, CDKN1A, CDKN1B, SRC, FOXO3, HIF1A, ACTB, EGFR, RELA, CASP9, CDC42, CCND1, CASP1, RAC1, PTPN1, PRKCD, STAT3, ESR1, PTK2, RHOA, ACTA2, KAT2B, PTPRC, FAS, CTNNB1, TP53, SQSTM1, BCL2L1 | 1.43998E-12 |
| RNA polymerase II-specific DNA-binding transcription factor binding | RB1, JUN, HDAC2, NR1I2, STAT3, MAPK14, FOXP3, HIF1A, SIRT1, ACTB, RELA, NFKBIA, CREB1, CTNNB1, PPARG, PPARA, TP53, PPARGC1A, NFE2L2 | 1.57518E-11 |
| Cytokine activity | IL10, TGFB2, EDN1, TGFB1, CXCL8, IL18, MIF, TNF, IL2, VEGFA, IL4, CXCL10, BMP2, IL6, IFNG, IL1B, IL12A | 3.09568E-10 |
| Growth factor activity | IL10, EGF, PDGFB, IGF2, FGF1, F2, PGF, AGT, IL2, VEGFA, IL4, IL6, IL12A | 3.98218E-10 |
| Phosphatase binding | CDKN1B, STAT3, PIK3R1, MAPK14, PTK2, EGFR, LGALS3, MAPK1, CTNNB1, TP53, JAK3, MET, MAPK3 | 1.14995E-08 |
| DNA-binding transcription factor binding | RB1, JUN, HDAC2, STAT3, FHL2, SIRT1, RELA, CREB1, MYC, DDIT3, CTNNB1, NLRP3, PPARG, PPARA, TP53, NFE2L2 | 3.16459E-08 |
| Growth factor receptor binding | IL4, IL10, IL6, SRC, EGF, IL1B, PDGFB, IL12A, FGF1, PGF, IL2, VEGFA | 4.48598E-08 |
| Protein tyrosine kinase activity | SRC, KIT, KDR, TEK, JAK3, MET, EPHB4, PTK2, FGFR2, EGFR, IGF1R, FGFR1 | 5.66818E-08 |

**Table S5.** Top 10 KEGG pathway enrichment analysis of overlapping targets of coronary artery bypass graft and sirolimus involved in neointimal hyperplasia

| **KEGG** | **Genes** | **Adjusted**  **p-value** |
| --- | --- | --- |
| Lipid and atherosclerosis | CXCL8, NCF1, NCF2, PIK3CD, PIK3CB, TNF, ICAM1, CASP9, CASP7, CASP8, AKT2, CASP3, CASP1, AKT1, OLR1, IL12A, RAC1, CD36, HRAS, HSP90AA1, MMP1, MMP3, IL18, CYBA, MMP9, RHOA, PIK3CA, DDIT3, IL1B, PPARG, TP53, SRC, PIK3R1, RELA, CDC42, MAPK8, NLRP3, MAPK1, MAPK3, ABCA1, XBP1, JUN, VCAM1, HSPA5, NOS3, STAT3, MAPK14, SELE, EIF2S1, PTK2, NFKB1, SELP, NFKBIA, MAPK10, IL6, FAS, BAX, NFE2L2, BCL2L1 | 5.50358E-66 |
| AGE-RAGE signaling pathway in diabetic complications | CXCL8, CDKN1B, SERPINE1, PIK3CD, PIK3R1, PIK3CB, TNF, RELA, ICAM1, CDC42, MAPK8, CCND1, AKT2, CASP3, AKT1, MAPK1, RAC1, HRAS, MAPK3, TGFB2, JUN, EDN1, TGFB1, VCAM1, PRKCB, NOS3, MMP2, PRKCD, STAT3, FN1, MAPK14, SELE, F3, NFKB1, AGT, VEGFA, MAPK10, IL6, COL1A2, PIK3CA, CDK4, IL1B, BAX | 2.07439E-57 |
| PI3K-Akt signaling pathway | CDKN1A, CDKN1B, IRS1, PTEN, PIK3CD, PIK3CB, FGF1, PIK3CG, IGF1R, CASP9, CCND1, AKT2, MYC, KDR, AKT1, RAC1, HRAS, JAK3, HSP90AA1, F2R, PGF, CREB1, CCNE2, PIK3CA, CCNE1, KIT, ITGA6, TP53, MET, PRKAA1, PDGFB, PIK3R1, FOXO3, EGFR, RELA, INS, SPP1, MAPK1, MCL1, MAPK3, NOS3, EGF, FN1, IGF2, IGF1, PTK2, IL2, NFKB1, MTOR, VEGFA, IL4, IL6, COL1A2, RPS6KB1, CDK4, IL2RA, CDK2, MDM2, TEK, FGFR2, FGFR1, BCL2L1 | 1.17381E-56 |
| Fluid shear stress and atherosclerosis | PRKAA1, NCF1, NCF2, SRC, PDGFB, PIK3CD, PIK3R1, PIK3CB, TNF,  ACTB, RELA, ICAM1, MAPK8, AKT2, KDR, AKT1, HMOX1, RAC1, JUN, EDN1, HSP90AA1, VCAM1, CAV3, NOS3, MMP2, CYBA, MAPK14, SELE, MMP9, NFKB1, PTK2, RHOA, VEGFA, MAPK10, PIK3CA, IFNG, IL1B, CTNNB1, SQSTM1, TP53, NFE2L2 | 6.97917E-47 |
| FoxO signaling pathway | CDKN1A, PRKAA1, CDKN1B, IRS1, PTEN, PIK3CD, PIK3R1, PIK3CB, FOXO3, EGFR, IGF1R, INS, MAPK8, CCND1, AKT2, S1PR1, AKT1, MAPK1, SKP2, HRAS, MAPK3, IL10, TGFB2, TGFB1, EGF, STAT3, IGF1, MAPK14, SIRT1, MAPK10, IL6, PIK3CA, CDK2, CAT, MDM2 | 2.54619E-38 |
| HIF-1 signaling pathway | CDKN1A, CDKN1B, SERPINE1, PIK3CD, PIK3R1, PIK3CB, ENO2, HIF1A, RELA, EGFR, IGF1R, INS, AKT2, NPPA, AKT1, HMOX1, MAPK1, MAPK3, EDN1, NOS2, PRKCB, EGF, NOS3, STAT3, IGF1, NFKB1, MTOR, VEGFA, IL6, PIK3CA, IFNG, RPS6KB1, TEK | 3.47966E-38 |
| TNF signaling pathway | PIK3CD, PIK3R1, PIK3CB, TNF, RELA, ICAM1, CASP7, MAPK8, CASP8, AKT2, CASP3, AKT1, MAPK1, MAPK3, JUN, EDN1, VCAM1, MMP3, MAPK14, SELE, MMP9, NFKB1, NFKBIA, MAPK10, CXCL10, IL6, MMP14, CREB1, PIK3CA, IL1B, FAS | 1.41853E-34 |
| Focal adhesion | SRC, PDGFB, PTEN, PIK3CD, PIK3R1, PIK3CB, ACTB, EGFR, MYLK, IGF1R, CDC42, MAPK8, CCND1, AKT2, KDR, SPP1, AKT1, MAPK1, RAC1, HRAS, MAPK3, JUN, CAV3, PRKCB, EGF, FN1, IGF1, PTK2, RHOA, PGF, VEGFA, MAPK10, COL1A2, PIK3CA, CTNNB1, ITGA6,  MET | 2.68411E-34 |
| Apoptosis | BECN1, PIK3CD, PIK3R1, PIK3CB, TNF, ACTB, CTSS, RELA, CASP9, CASP7, MAPK8, CASP8, AKT2, CTSK, CASP3, LMNA, AKT1, MAPK1, HRAS, MCL1, MAPK3, JUN, EIF2S1, NFKB1, NFKBIA, MAPK10, PIK3CA, DDIT3, BIRC5, FAS, BAX, TP53, BCL2L1 | 3.87485E-34 |
| C-type lectin receptor signaling pathway | SRC, PIK3CD, PIK3R1, PIK3CB, TNF, RELA, MAPK8, CASP8, AKT2, CASP1, AKT1, NLRP3, MAPK1, IL12A, HRAS, MAPK3, IL10, JUN, PRKCD, MAPK14, NFKB1, IL2, RHOA, NFKBIA, MAPK10, IL6, PIK3CA, IL1B, MDM2 | 1.29265E-32 |

**Table S6.** Top 10 biological process enrichment analysis of overlapping targets of coronary artery bypass graft and rosuvastatin involved in neointimal hyperplasia

| **Biological process** | **Genes** | **Adjusted p-value** |
| --- | --- | --- |
| Negative regulation of apoptotic process | ITGB1, HDAC2, SRC, PIK3R1, TNF, EGFR, IGF1R, MAPK8, AKT2, CASP3, KDR, AKT1, MAPK1, CCL2, CTSG, MCL1, CSNK2A1, MIF, IGF1, MMP9, PTK2, IL2, SOD1, ALB, CAT, TEK | 1.89715E-15 |
| Regulation of inflammatory response | PTGIS, CMA1, MMP3, APOA1, NR1H3, TNF, SELE, MMP9, IL2, ACE2, IL1B, AGTR1, CASP1, PPARG, TEK, PPARA, S100A9, ELANE | 3.62003E-14 |
| Extracellular matrix disassembly | MMP12, MMP14, MMP7, MMP1, CMA1, CTSK, MMP2, MMP3, CTSG, MMP9, CTSS, ELANE | 3.86799E-13 |
| Extracellular matrix organization | ITGB1, TGFB1, MMP7, ITGA4, MMP1, CMA1, ITGB3, MMP2, MMP3, MMP9, CTSS, MMP12, MMP14, TTR, CTSK, KDR, PECAM1, CTSG, ELANE | 1.41623E-12 |
| Cellular response to oxygen-containing compound | P2RY12, DAPK1, MMP3, NR1H3, MAPK14, ESR1, MMP9, EGFR, MAPK8, IL1B, CDK2, CASP1, AKT1, MAPK1, CCL2, CTSG, TGM2 | 4.59619E-10 |
| Cellular response to oxidative stress | MAPK8, DAPK1, CAT, AKT1, HMOX1, MAPK1, CYBA,  MMP9, EGFR, NFE2L2, SOD1 | 7.69466E-09 |
| Vascular endothelial growth factor receptor signaling pathway | HSP90AA1, SRC, ITGB3, KDR, CYBA, PIK3R1, MAPK14, PGF, PTK2 | 7.69466E-09 |
| Extracellular structure organization | ITGB1, MMP7, ITGA4, MMP1, ITGB3, MMP2, MMP3, MP9, MMP12, MMP14, TTR, KDR, PECAM1 | 1.70767E-08 |
| Regulation of blood vessel endothelial cell migration | TGFB1, NOS3, KDR, AKT1, PPARG, MAPK14, TNF, FR1 | 3.62269E-08 |
| Platelet degranulation | SELP, TGFB1, SERPINA1, ITGB3, ALB, PECAM1, APOA1, IGF1, SOD1 | 1.29722E-06 |

**Table S7.** Top 10 cellular component enrichment analysis of overlapping targets of coronary artery bypass graft and rosuvastatin involved in neointimal hyperplasia

| **Cellular component** | **Genes** | **Adjusted p-value** |
| --- | --- | --- |
| Secretory granule lumen | HSP90AA1, TGFB1, SERPINA1, ARG1, APOA1, MIF,  IGF1, MAPK14, TTR, ALB, CAT, LCN2, MAPK1, CTSG, S100A9, ELANE | 9.29312E-09 |
| Membrane raft | ABCA1, ITGB1, ACE2, MME, SRC, KDR, TEK, TNF, SELE, EGFR | 2.95561E-06 |
| Collagen-containing extracellular matrix | TGFB1, SERPINA1, CMA1, MMP2, APOA1, F2, MMP9, CTSS, LGALS3, CTSG, S100A9, ELANE, FGFR2, TGM2 | 2.95561E-06 |
| Vacuolar lumen | HSP90AA1, TTR, ARG1, CTSK, MAPK1, CTSG, APOB, ELANE, CTSS | 1.16247E-05 |
| Focal adhesion | PDGFRB, ITGB1, MME, ITGA4, SRC, ITGB3, PTK2, EGFR, MMP14, PLAU, CAT, MAPK1, TGM2 | 1.16247E-05 |
| Cell-substrate junction | PDGFRB, ITGB1, MME, ITGA4, SRC, ITGB3, PTK2, EGFR, MMP14, PLAU, CAT, MAPK1, TGM2 | 1.16247E-05 |
| Ficolin-1-rich granule lumen | HSP90AA1, SERPINA1, CAT, MAPK1, MIF, MAPK14, MMP9, CTSS | 1.16247E-05 |
| Platelet alpha granule | SELP, SERPINA1, TGFB1, ITGB3, ALB, PECAM1, IGF1 | 1.59172E-05 |
| Ficolin-1-rich granule | LGALS3, HSP90AA1, SERPINA1, CAT, MAPK1, MIF, MAPK14, MMP9, CTSS | 1.90186E-05 |
| Endocytic vesicle | ABCA1, ACE2, HSP90AA1, NOS3, MDM2, APOA1, APOB, CTSS | 0.00019612 |

**Table S8.** Top 10 molecular function enrichment analysis of overlapping targets of coronary artery bypass graft and rosuvastatin involved in neointimal hyperplasia

| **Molecular function** | **Genes** | **Adjusted p-value** |
| --- | --- | --- |
| Endopeptidase activity | C1S, ECE1, CTSS, CASP7, CASP8, PLAU, CTSK, CASP3, CASP1, CTSG, ELANE, ACE, MMP7, MME, F10, MMP1, CMA1, MMP2, MMP3, F2, MMP9, MMP12, ACE2, MMP14, REN | 8.29395E-19 |
| Protein tyrosine kinase activity | PDGFRB, SRC, PTK2, EGFR, IGF1R, KIT, KDR, TEK, JAK3, MET, EPHB4, FGFR2, FGFR1 | 1.10851E-11 |
| Transmembrane receptor protein kinase activity | PDGFRB, KIT, KDR, TEK, MET, EPHB4, EGFR, FGFR2, IGF1R, FGFR1 | 1.60499E-10 |
| Transmembrane receptor protein tyrosine kinase activity | PDGFRB, KIT, KDR, TEK, MET, EPHB4, EGFR, FGFR2, IGF1R, FGFR1 | 1.60499E-10 |
| Metallopeptidase activity | MMP12, ACE2, MMP14, ACE, MMP7, MME, MMP1, MMP2, MMP3, ECE1, MMP9 | 8.80195E-09 |
| Metalloendopeptidase activity | MMP12, MMP14, MMP7, MME, MMP1, MMP2, MMP3, ECE1, MMP9 | 6.74608E-08 |
| Transition metal ion binding | PTPN1, ACE, MME, ARG1, NOS3, ECE1, CYP3A4, MMP9, SOD1, MMP12, ACE2, ALB, MDM2, LCN2, PPARG, S100A9 | 3.76116E-07 |
| Serine-type endopeptidase activity | C1S, F10, PLAU, CMA1, MMP2, CTSG, F2, MMP9, ELANE | 4.62887E-07 |
| Serine-type peptidase activity | C1S, F10, PLAU, CMA1, MMP2, CTSG, F2, MMP9, ELANE | 1.89587E-06 |
| Heme binding | CYP26A1, PTGIS, NOS2, SRC, CBS, NOS3, CAT, HMOX1 | 2.01525E-06 |

**Table S9.** Top 10 KEGG pathway enrichment analysis of overlapping targets of coronary artery bypass graft and rosuvastatin involved in neointimal hyperplasia

| **KEGG** | **Genes** | **Adjusted p-value** |
| --- | --- | --- |
| Lipid and atherosclerosis | SRC, PIK3R1, TNF, CASP7, MAPK8, CASP8, AKT2, CASP3, CASP1, AKT1, MAPK1, CCL2, APOB, HRAS, ABCA1, HSP90AA1, MMP1, NOS3, MMP3, APOA1, CYBA, MAPK14, SELE, MMP9, PTK2, SELP, MAPK10, IL1B, BAX, PPARG, NFE2L2 | 1.88656E-32 |
| Fluid shear stress and atherosclerosis | HSP90AA1, SRC, NOS3, ITGB3, MMP2, CYBA, PIK3R1, MAPK14, TNF, SELE, MMP9, PTK2, MAPK10, MAPK8, AKT2, IL1B, KDR, PECAM1, AKT1, HMOX1, CCL2, NFE2L2 | 1.33573E-23 |
| PI3K-Akt signaling pathway | ITGB1, ITGB3, PIK3R1, FGF1, PIK3CG, EGFR, IGF1R, AKT2, KDR, AKT1, MAPK1, HRAS, JAK3, MCL1, PDGFRB, HSP90AA1 ITGA4, NOS3, IGF1, PGF, PTK2, IL2, CDK2, KIT, MDM2, TEK, MET, FGFR2, FGFR1 | 2.84561E-23 |
| AGE-RAGE signaling pathway in diabetic complications | TGFB1, NOS3, MMP2, PIK3R1, MAPK14, TNF, SELE, MAPK10, MAPK8, AKT2, CASP3, IL1B, AGTR1, AKT1,  CCL2, MAPK1, BAX, HRAS | 1.79973E-20 |
| Rap1 signaling pathway | PDGFRB, ITGB1, SRC, ITGB3, PIK3R1, IGF1, FGF1, MAPK14, PGF, EGFR, IGF1R, AKT2, KIT, KDR, AKT1, MAPK1, TEK, HRAS, MET, FGFR2, FGFR1 | 1.36615E-18 |
| TNF signaling pathway | MMP3, PIK3R1, MAPK14, TNF, SELE, MMP9, MAPK10, CASP7, MMP14, MAPK8, CASP8, AKT2, CASP3, IL1B, AKT1, CCL2, MAPK1 | 3.85867E-18 |
| MAPK signaling pathway | PDGFRB, TGFB1, IGF1, FGF1, MAPK14, TNF, PGF, EGFR,IGF1R, MAPK10, MAPK8, AKT2, CASP3, IL1B, KIT, KDR, AKT1, MAPK1, TEK, HRAS, MET, FGFR2, FGFR1 | 4.19402E-18 |
| Relaxin signaling pathway | TGFB1, NOS2, MMP1, SRC, NOS3, MMP2, PIK3R1, MAPK14, MMP9, EGFR, MAPK10, MAPK8, EDNRB, AKT2, AKT1, MAPK1, HRAS | 3.83237E-17 |
| Focal adhesion | PDGFRB, ITGB1, ITGA4, SRC, ITGB3, PIK3R1, IGF1, PGF, PTK2, EGFR, IGF1R, MAPK10, MAPK8, AKT2, KDR, AKT1, MAPK1, HRAS, MET | 1.67561E-16 |
| IL-17 signaling pathway | HSP90AA1, MMP1, MMP3, MAPK14, TNF, MMP9, MAPK10, MAPK8, CASP8, CASP3, IL1B, LCN2, CCL2, MAPK1, S100A9 | 1.72105E-16 |

**Table S10.** Top 10 biological process enrichment analysis of overlapping targets of coronary artery bypass graft and both drugs involved in neointimal hyperplasia

| **Biological process** | **Genes** | **Adjusted p-value** |
| --- | --- | --- |
| Negative regulation of apoptotic process | HDAC2, SRC, MIF, PIK3R1, IGF1, TNF, MMP9, EGFR, PTK2, IL2, IGF1R, SOD1, MAPK8, CASP3, AKT2, CAT, ALB, KDR, AKT1, MAPK1, CTSG, TEK, MCL1 | 1.66895E-14 |
| Extracellular matrix disassembly | MMP12, MMP14, MMP7, MMP1, CMA1, CTSK, MMP2, MMP3, CTSG, MMP9, CTSS, ELANE | 4.26476E-14 |
| Regulation of inflammatory response | CMA1, MMP3, NR1H3, TNF, MMP9, SELE, IL2, ACE2, IL1B, CASP1, PPARG, TEK, PPARA, S100A9, ELANE | 4.82227E-12 |
| Cellular response to oxygen-containing compound | P2RY12, DAPK1, MMP3, NR1H3, MAPK14, MMP9, ESR1, EGFR, MAPK8, IL1B, CDK2, CASP1, AKT1, MAPK1, CTSG, TGM2 | 1.86511E-10 |
| Extracellular matrix organization | MMP7, TGFB1, MMP1, CMA1, MMP2, MMP3, MMP9, CTSS, MMP12, MMP14, TTR, CTSK, KDR, CTSG, ELANE | 6.91047E-10 |
| Cellular response to oxidative stress | MAPK8, DAPK1, CAT, AKT1, HMOX1, MAPK1, CYBA, MMP9, EGFR, SOD1, NFE2L2 | 9.73019E-10 |
| Regulation of blood vessel endothelial cell migration | TGFB1, NOS3, KDR, AKT1, PPARG, MAPK14, TNF, FGFR1 | 8.38921E-09 |
| Vascular endothelial growth factor receptor signaling pathway | HSP90AA1, SRC, KDR, CYBA, PIK3R1, MAPK14, PGF, PTK2 | 3.79661E-08 |
| Regulation of nitric-oxide synthase activity | HSP90AA1, NOS3, IL1B, AKT1, TNF, EGFR | 8.32987E-07 |
| Extracellular structure organization | MMP12, MMP14, MMP7, TTR, MMP1, MMP2, MMP3, KDR, MMP9 | 1.60149E-05 |

**Table S11.** Top 10 cellular component enrichment analysis of overlapping targets of coronary artery bypass graft and both drugs involved in neointimal hyperplasia

| **Cellular component** | **Genes** | **Adjusted p-value** |
| --- | --- | --- |
| Secretory granule lumen | HSP90AA1, SERPINA1, TGFB1, MIF, IGF1, MAPK14, TTR, CAT, ALB, LCN2, MAPK1, CTSG, S100A9, ELANE | 4.40212E-08 |
| Collagen-containing extracellular matrix | SERPINA1, TGFB1, CMA1, MMP2, F2, MMP9, CTSS, LGALS3, CTSG, S100A9, ELANE, FGFR2, TGM2 | 2.16527E-06 |
| Membrane raft | ABCA1, ACE2, MME, SRC, KDR, TEK, TNF, SELE, EGFR | 3.56783E-06 |
| Ficolin-1-rich granule lumen | HSP90AA1, SERPINA1, CAT, MAPK1, MIF, MAPK14, MMP9, CTSS | 3.56783E-06 |
| Ficolin-1-rich granule | LGALS3, HSP90AA1, SERPINA1, CAT, MAPK1, MIF, MAPK14, MMP9, CTSS | 5.47928E-06 |
| Vacuolar lumen | HSP90AA1, TTR, CTSK, MAPK1, CTSG, CTSS, ELANE | 0.000204203 |
| Platelet alpha granule | SELP, SERPINA1, TGFB1, ALB, IGF1 | 0.000949449 |
| Focal adhesion | MMP14, MME, PLAU, SRC, CAT, MAPK1, EGFR, PTK2, TGM2 | 0.001205216 |
| Cell-substrate junction | MMP14, MME, PLAU, SRC, CAT, MAPK1, EGFR, PTK2, TGM2 | 0.001265156 |
| Caveola | SRC, NOS3, MAPK1, SELE | 0.001676182 |

**Table S12.** Top 10 molecular function enrichment analysis of overlapping targets of coronary artery bypass graft and both drugs involved in neointimal hyperplasia

| **Molecular function** | **Genes** | **Adjusted p-value** |
| --- | --- | --- |
| Endopeptidase activity | MMP7, MME, C1S, F10, MMP1, CMA1, MMP2, MMP3, F2, MMP9, CTSS, MMP12, ACE2, CASP7, MMP14,CASP8, PLAU, CTSK, CASP3, CASP1, CTSG, REN, ELANE | 1.19552E-18 |
| Protein tyrosine kinase activity | SRC, KIT, KDR, TEK, JAK3, MET, EPHB4, EGFR, PTK2, FGFR2, IGF1R, FGFR1 | 1.79289E-11 |
| Transmembrane receptor protein kinase activity | KIT, KDR, TEK, MET, EPHB4, EGFR, FGFR2, IGF1R, FGFR1 | 7.09973E-10 |
| Transmembrane receptor protein tyrosine kinase activity | KIT, KDR, TEK, MET, EPHB4, EGFR, FGFR2, IGF1R, FGFR1 | 7.09973E-10 |
| Serine-type endopeptidase activity | C1S, F10, PLAU, CMA1, MMP2, CTSG, F2, MMP9, ELANE | 9.67702E-08 |
| Metalloendopeptidase activity | MMP12, MMP14, MMP7, MME, MMP1, MMP2, MMP3, MMP9 | 2.36993E-07 |
| Metallopeptidase activity | MMP12, ACE2, MMP14, MMP7, MME, MMP1, MMP2, MMP3, MMP9 | 2.44033E-07 |
| Serine-type peptidase activity | C1S, F10, PLAU, CMA1, MMP2, CTSG, F2, MMP9, ELANE | 2.84508E-07 |
| Phosphatase binding | LGALS3, MAPK1, PIK3R1, MAPK14, JAK3, MET, EGFR, PTK2 | 2.15221E-06 |
| Protein phosphatase binding | LGALS3, PIK3R1, MAPK14, JAK3, MET, EGFR, PTK2, SOD1 | 3.49922E-06 |

**Table S13.** Top 10 KEGG pathway enrichment analysis of overlapping targets of coronary artery bypass graft and both drugs involved in neointimal hyperplasia

| **KEGG** | **Genes** | **Adjusted p-value** |
| --- | --- | --- |
| Lipid and atherosclerosis | SRC, PIK3R1, TNF, CASP7, MAPK8, CASP8, CASP3, AKT2, CASP1, AKT1, MAPK1, HRAS, ABCA1, HSP90AA1, MMP1, NOS3, MMP3, CYBA, MAPK14, MMP9, SELE, PTK2, MAPK10, SELP, IL1B, BAX, PPARG, NFE2L2 | 9.65327E-31 |
| Fluid shear stress and atherosclerosis | HSP90AA1, SRC, NOS3, MMP2, CYBA, PIK3R1, MAPK14, TNF, MMP9, SELE, PTK2, MAPK10, MAPK8, AKT2, IL1B, KDR, AKT1, HMOX1, NFE2L2 | 5.56189E-21 |
| PI3K-Akt signaling pathway | PIK3R1, FGF1, PIK3CG, EGFR, IGF1R, AKT2, KDR, AKT1, MAPK1, HRAS, JAK3, MCL1, HSP90AA1, NOS3, IGF1, PGF, PTK2, IL2, KIT, CDK2, MDM2, TEK, MET, FGFR2, FGFR1 | 7.26477E-21 |
| AGE-RAGE signaling pathway in diabetic complications | TGFB1, NOS3, MMP2, PIK3R1, MAPK14, TNF, SELE, MAPK10, MAPK8, CASP3, AKT2, IL1B, AKT1, MAPK1, BAX, HRAS | 6.10025E-19 |
| MAPK signaling pathway | TGFB1, IGF1, FGF1, MAPK14, TNF, PGF, EGFR, IGF1R, MAPK10, MAPK8, CASP3, AKT2, IL1B, KIT, KDR, AKT1, MAPK1, TEK, HRAS, MET, FGFR2, FGFR1 | 6.10025E-19 |
| Relaxin signaling pathway | TGFB1, NOS2, MMP1, SRC, NOS3, MMP2, PIK3R1, MAPK14, MMP9, EGFR, MAPK10, MAPK8, EDNRB, AKT2, AKT1, MAPK1, HRAS | 9.81145E-19 |
| TNF signaling pathway | MMP3, PIK3R1, MAPK14, TNF, MMP9, SELE, MAPK10, CASP7, MMP14, MAPK8, CASP8, CASP3, AKT2, IL1B, AKT1, MAPK1 | 3.137E-18 |
| C-type lectin receptor signaling pathway | SRC, PIK3R1, MAPK14, TNF, IL2, MAPK10, MAPK8, CASP8, AKT2, IL1B, CASP1, MDM2, AKT1, MAPK1, HRAS | 3.61244E-17 |
| Rap1 signaling pathway | SRC, PIK3R1, IGF1, FGF1, MAPK14, PGF, EGFR, IGF1R, AKT2, KIT, KDR, AKT1, MAPK1, TEK, HRAS, MET, FGFR2, FGFR1 | 1.31573E-16 |
| IL-17 signaling pathway | HSP90AA1, MMP1, MMP3, MAPK14, TNF, MMP9, MAPK10, MAPK8, CASP8, CASP3, IL1B, LCN2, MAPK1, S100A9 | 2.58281E-16 |

**Table S14.** Top 10 significant bottleneck genes ranked by CytoHubba

| **No.** | **Name** | **Description** | **MCC^1)^ Score** | **BottleNeck Score** | **DC^2)^** | **BC^3)^** | **CC^4)^** |
| --- | --- | --- | --- | --- | --- | --- | --- |
| **Sirolimus** | | | | | | | |
| 1 | TP53^*5)^ | Tumor protein p53 | 9.22E+13 | 50.00 | 146.00 | 972.93 | 178.00 |
| 2 | IL6^*^ | Interleukin 6 | 9.22E+13 | 19.00 | 163.00 | 1400.41 | 186.50 |
| 3 | STAT3^*^ | Signal transducer and activator of transcription 3 | 9.22E+13 | 15.00 | 134.00 | 541.92 | 172.00 |
| 3 | VCAM1 | Vascular cell adhesion molecule 1 | 9.22E+13 | 15.00 | 87.00 | 194.11 | 148.33 |
| 5 | IL1B | Interleukin 1 beta | 9.22E+13 | 8.00 | 146.00 | 898.27 | 178.00 |
| 6 | MAPK14 | Mitogen-activated protein kinase 14 | 9.22E+13 | 6.00 | 93.00 | 214.59 | 151.50 |
| 6 | ESR1 | Estrogen receptor 1 | 9.22E+13 | 6.00 | 113.00 | 489.63 | 161.33 |
| 6 | ALB^*^ | Albumin | 9.22E+13 | 6.00 | 161.00 | 1908.35 | 185.50 |
| 9 | AKT1 | AKT serine/threonine kinase 1 | 9.22E+13 | 4.00 | 173.00 | 1687.52 | 191.50 |
| 9 | MMP2 | Matrix metallopeptidase 2 | 9.22E+13 | 5.00 | 99.00 | 220.08 | 154.50 |
| 9 | SRC | SRC proto-oncogene, non-receptor tyrosine kinase | 9.22E+13 | 5.00 | 134.00 | 766.75 | 172.00 |
| **Rosuvastatin** | | | | | | | |
| 1 | ALB^*^ | Albumin | 9.22E+13 | 44.00 | 93.00 | 1177.20 | 105.08 |
| 2 | AKT1^*^ | AKT serine/threonine kinase 1 | 9.22E+13 | 11.00 | 89.00 | 878.05 | 103.08 |
| 3 | TNF^*^ | Tumor necrosis factor | 9.22E+13 | 8.00 | 97.00 | 1270.34 | 106.92 |
| 4 | IL1B^*^ | Interleukin 1 beta | 9.22E+13 | 6.00 | 79.00 | 557.01 | 97.92 |
| 5 | SELE | Selectin E | 1.82E+13 | 5.00 | 36.00 | 94.79 | 76.83 |
| 6 | CAT | Catalase | 9.22E+13 | 4.00 | 48.00 | 194.11 | 82.58 |
| 6 | PTGIS | Prostaglandin I2 synthase | 279 | 5.00 | 12.00 | 400.50 | 61.67 |
| 8 | MDM2 | MDM2 proto-oncogene | 9.22E+13 | 2.00 | 33.00 | 50.64 | 74.42 |
| 8 | ESR1 | Estrogen receptor 1 | 9.22E+13 | 3.00 | 57.00 | 260.82 | 86.92 |
| 8 | CASP3^*^ | Caspase 3 | 9.22E+13 | 3.00 | 65.00 | 379.67 | 90.92 |
| 8 | PECAM1 | Platelet and endothelial cell adhesion molecule 1 | 9.22E+13 | 1.00 | 50.00 | 129.10 | 83.42 |
| 8 | MMP9^*^ | Matrix metallopeptidase 9 | 9.22E+13 | 5.00 | 77.00 | 474.21 | 96.92 |
| **Sirolimus + Rosuvastatin** | | | | | | | |
| 1 | ALB^*^ | Albumin | 9.22E+13 | 40.00 | 79.00 | 992.34 | 87.00 |
| 2 | AKT1^*^ | AKT serine/threonine kinase 1 | 9.22E+13 | 11.00 | 75.00 | 629.42 | 85.00 |
| 3 | TNF^*^ | Tumor necrosis factor | 9.22E+13 | 5.00 | 82.00 | 952.30 | 88.50 |
| 3 | ESR1 | Estrogen receptor 1 | 9.22E+13 | 5.00 | 49.00 | 194.33 | 71.83 |
| 5 | MMP9^*^ | Matrix metallopeptidase 9 | 9.22E+13 | 3.00 | 66.00 | 404.38 | 80.50 |
| 5 | IL1B | Interleukin 1 beta | 9.22E+13 | 7.00 | 67.00 | 418.99 | 81.00 |
| 7 | IGF1 | Insulin like growth factor 1 | 9.22E+13 | 3.00 | 54.00 | 190.52 | 74.50 |
| 7 | CAT | Catalase | 9.22E+13 | 3.00 | 38.00 | 110.59 | 66.50 |
| 7 | CASP3^*^ | Caspase 3 | 9.22E+13 | 3.00 | 58.00 | 322.68 | 76.50 |
| 10 | SRC^*^ | SRC proto-oncogene, non-receptor tyrosine kinase | 9.22E+13 | 2.00 | 57.00 | 227.32 | 76.00 |
| 10 | PPARA | Peroxisome proliferator activated receptor alpha | 2.99E+12 | 1.00 | 31.00 | 85.01 | 63.00 |
| 10 | HSP90AA1^*^ | Heat shock protein 90 alpha family class A member 1 | 9.22E+13 | 2.00 | 48.00 | 162.96 | 71.50 |
| 10 | HMOX1 | Heme oxygenase 1 | 3.46E+12 | 2.00 | 34.00 | 228.22 | 64.50 |
| 10 | MDM2 | MDM2 proto-oncogene | 9.22E+13 | 2.00 | 31.00 | 37.13 | 62.83 |
| 10 | SERPINA1 | Serpin family A member 1 | 1670018 | 2.00 | 22.00 | 70.77 | 58.33 |
| 10 | MMP2 | Matrix metallopeptidase 2 | 7.18E+13 | 2.00 | 47.00 | 127.75 | 71.00 |

^1)^ MCC, maximal clique centrality; ^2)^ DC, degree centrality; ^3)^ BC, betweenness centrality; ^4)^ CC, closeness centrality; ^5)^ Asterisks (*) indicate genes with overlapping hub-bottlenecks.

**Fig. S1.** Local drug delivery using a localized perivascular drug delivery device.

(A) Diagram of the localized perivascular drug delivery device with sequential release of sirolimus and rosuvastatin. (B) *In vitro* test of the local drug delivery device. Fluorescence image of a spot inserted into an agarose gel after use of the localized perivascular drug delivery device. (C) Optical image and (D) scanning electron transmission image of the localized perivascular drug delivery device.

**Fig. S2.** *In vivo* safety assessment of the localized perivascular drug delivery device.

(A) Changes in body weight of the rabbits in each group during the 4-week experiment (n = 3–5/group). (B–D) Toxicity assessment of the localized perivascular drug delivery device via H&E staining in organs (liver, kidney, and skeletal muscle) of rabbits in the control and SIR+RSV groups. No significant pathological differences were observed between the two groups (scale bars = 100 μm). H&E, hematoxylin and eosin; SIR+RSV, combination of sirolimus and rosuvastatin.

**Fig. S3.** Effect of the localized perivascular drug delivery device on cell morphology.

(A and B) Transmission electron microscopy sections of rabbit abdominal aortas at both the hyper-acute stage (1 week) and the acute stage (2 weeks) after injury. The aortas were treated with a localized perivascular drug delivery device containing either no drug (control) or SIR+RSV. SIR+RSV, combination of sirolimus and rosuvastatin.

**Fig. S4.** Full-length western blot images.

(A-C) Full-length western blot image for Figures 8A, 9A, and 10A. SIR+RSV, combination of sirolimus and rosuvastatin. IL-6, interleukin 6; TNF-α, tumor necrosis factor alpha; IL-1β, interleukin 1 beta; STAT3, signal transducer and activator of transcription 3; p-STAT3, phosphorylated STAT3; Akt, a protein cloned from the v-akt oncogene of retrovirus AKT8; mTOR, mammalian target of rapamycin; p-mTOR, phosphorylated mTOR; NF-κB, nuclear factor kappa-light-chain-enhancer of activated B cells; MMP, matrix metalloproteinase.
